# *Eriobotrya japonica* (Loquat) Leaf Extract: An Integral In Vitro and In Ovo Approach to Antimelanoma Potential and Safety

**DOI:** 10.3390/ijms27177676

**Published:** 2026-08-27

**Authors:** Andreea Maria Cristea, Ioana Gabriela Macaşoi, Diana Haj Ali, Raluca Andreea Jupâneanț, Dorina Elena Coricovac, Cristina Adriana Dehelean, Diana-Simona Tchiakpe-Antal, Victor Dumitrașcu, Alex-Robert Jîjie, Mihaela Lăcătuș, Alina Anton

**Affiliations:** 1 University Clinic of Toxicology, Drug Industry, Management and Legislation, Faculty of Pharmacy, “Victor Babes” University of Medicine and Pharmacy, 2nd Eftimie Murgu Square, 300041 Timisoara, Romania; 2 Research Centre for Pharmaco-Toxicological Evaluation, Faculty of Pharmacy, “Victor Babes” University of Medicine and Pharmacy, 2nd Eftimie Murgu Square, 300041 Timisoara, Romania; 3 Doctoral School, “Victor Babes” University of Medicine and Pharmacy, 2nd Eftimie Murgu Square, 300041 Timisoara, Romania; 4 Biochemistry and Pharmacology Department, Discipline of Pharmacology, Faculty of Medicine, “Victor Babes” University of Medicine and Pharmacy, 2nd Eftimie Murgu Square, 300041 Timișoara, Romania; 5 Department of Pharmaceutical Botany, Faculty of Pharmacy, “Victor Babes” University of Medicine and Pharmacy Timisoara, 2nd Eftimie Murgu Square, 300041 Timisoara, Romania; 6 Doctoral School “Engineering of Vegetable and Animal Resources”, University of Life Sciences “King Mihai I”, Aradului Street 119, 300645 Timisoara, Romania

**Keywords:** *Eriobotrya japonica*, antimelanoma activity, oxidative stress, apoptosis-like cell death, phenolic compounds, irritant potential, HET-CAM

## Abstract

Melanoma continues to be one of the most challenging diseases in oncology, given its aggressiveness and increased resistance to existing therapies. Therefore, the identification of novel therapeutic strategies is of considerable interest, with medicinal plants representing a promising source of bioactive compounds. In the present study, the phytochemical profile and the in vitro biological activity of a lyophilized aqueous leaf extract of *Eriobotrya japonica* (Thunb.) Lindl. cultivated in Romania were investigated. The extract was characterized by determining the total phenolic content, antioxidant capacity, and polyphenolic composition using UHPLC-MS/MS. Its cytotoxic potential and cytocompatibility were evaluated at concentrations of 50, 100, 200, 300, 400, and 500 µg/mL in two human melanoma cell lines (RPMI 7951 and A375) and in the human keratinocyte cell line HaCaT. Cell viability and morphological alterations were assessed in all three cell lines. Subsequently, the study focused on assessing the effects of the extract on melanoma cells by evaluating lysosome integrity and the intracellular production of reactive oxygen species (ROS). The impact on mitochondrial membrane potential, as well as on mitochondrial and nuclear morphology, was also determined. Finally, the HET-CAM test was carried out to assess the extract’s preliminary irritant potential at 500 µg/mL. The results showed that the extract presented a high content of phenolic compounds and high antioxidant capacity. The most abundant compounds in the extract were chlorogenic acid, rutin, 3,5-dihydroxybenzoic acid/3,4-dihydroxybenzoic acid, and p-coumaric acid/trans-p-coumaric acid. With regard to biological activity, the treatment induced low cytotoxicity in HaCaT cells including at 500 µg/mL. At the same time, it produced a dose-dependent reduction in the viability of melanoma cells, accompanied by impaired lysosomal integrity. It was observed that the extract increased ROS levels and mitochondrial membrane depolarization, thereby inducing morphological changes at the mitochondrial and nuclear levels. However, across all performed assays, the most pronounced effects were observed starting at a concentration of 300 µg/mL. The HET-CAM assay classified the extract as non-irritant at 500 µg/mL. These findings suggest that *Eriobotrya japonica* leaf extract exerts antimelanoma effects associated with oxidative stress and mitochondrial dysfunction while maintaining a favorable preliminary safety profile.

## 1. Introduction

Skin cancer is considered to be one of the most commonly diagnosed forms of cancer worldwide. Malignant melanoma, which belongs to this category of cancers, is continuing to experience a growing number of newly diagnosed cases [1]. However, melanoma continues to be the most aggressive form of skin cancer. Moreover, the prognosis depends heavily on the timing of diagnosis, as in the advanced stages of the disease the risk of metastasis is increased, and the survival rate decreases accordingly [2]. Advanced therapies developed in recent years, such as targeted drugs and immune checkpoint inhibitor therapies, have improved survival rates in metastatic melanoma [3]. Despite this, the long-term clinical value of these treatments is often limited by the development of resistance, unsatisfactory response rates in some cases, and the adverse effects linked to the therapy, which highlights the ongoing need for alternative and complementary therapeutic options [4]. Resistance to melanoma treatments is one of the reasons for treatment failure in the advanced stages. One of the main causes of treatment resistance is the genetic and phenotypic heterogeneity of the tumor, which allows cancer cells to adapt by reactivating signaling pathways or by activating alternative survival mechanisms. Thus, cellular heterogeneity allows tumor populations with unique biological characteristics to coexist, some of which may exhibit inherent resistance to treatment, thereby contributing to the persistence of the disease. Consequently, many patients exhibit only temporary or incomplete remissions, which will eventually lead to disease recurrence [5,6].

Taking into account the limitations of conventional cancer treatments, growing attention has shifted towards plant-derived compounds. These compounds provide structural diversity and may present enhanced safety profiles in comparison to synthetic drugs [7]. This interest is warranted by the outstanding chemical diversity of natural products. Therefore, over the years, plants constitute a valuable supply of bioactive compounds and have substantially contributed to the discovery of anticancer drugs currently used in clinical practice [8].

Among phytochemicals, substances with antioxidant activity, such as polyphenols and flavonoids, have been shown to possess significant anticancer activity, particularly in the case of tumors associated with increased intracellular oxidative stress, including melanoma [9]. Throughout plant species, *Rosaceae* family species are among those rich in compounds with antioxidant properties [10]. Furthermore, various species belonging to the *Rosaceae* family have been shown to have pro-apoptotic and antiproliferative effects in preclinical studies, suggesting their potential to be explored as anticancer agents.

In recent years, particular scientific attention has been allocated to *Eriobotrya japonica* (Thunb.) Lindl. (loquat), member of the *Rosaceae* family, an evergreen, subtropical fruit tree that has long been used for its therapeutic qualities [11]. Loquat is a notable reservoir of bioactive compounds, including phenolic acids, flavonoids, triterpenoids, carotenoids, vitamins, minerals, and organic acids, compounds that exert a myriad of pharmacological activities, such as antioxidant, anti-inflammatory, hypoglycemic, antiviral, cytotoxic, anticancer, hypolipidemic, neuroprotective, cardioprotective, hepatoprotective, and antimicrobial effects [12,13]. Moreover, the antitumor potential of loquat extracts was proven in different cancer cell lines (liver—HepG2, lung—A549, prostate—Pca, breast—MCF-7 and MDA-MB-231, colon—Caco-2 and HT-29, glioblastoma—U87MG, leukemia cells, and oral tumor cells) and preclinical mouse models of chemical induced skin carcinoma and breast adenocarcinoma [12,13,14,15,16]. Even though the extract obtained from the loquat fruits has a high amount of phytoconstituents with biological effects, it was shown that the leaves extract displayed an enhanced antioxidant and anticancer impact compared to the fruit extract [13]. The leaves of loquat have long been used in traditional Chinese medicine for the treatment of pain, inflammation, asthma, diabetes, and cancer, and multiple studies have gathered data that support the antidiabetic, anti-obesity, anti-inflammatory, and anticancer effects [17,18]. However, to the best of our knowledge, the chemical composition of the aqueous leaf extract of loquat cultivar grown in Romania has not been investigated so far, and the antimelanoma potential of this extract is also underexplored.

Hence, in the present study, in order to identify the phytochemical profile in loquat (Ej) aqueous leaf extract and to explore its antimelanoma potential, a detailed physicochemical characterization was conducted, along with an in vitro toxicological screening in two human melanoma cell lines (RPMI 7951 and A375). The parameters investigated were cell viability, cytotoxicity, morphological alterations, ROS (reactive oxygen species) production, mitochondrial membrane potential, mitochondria integrity, and nuclear changes. In addition, the safety profile of the extract in human keratinocytes (HaCaT) and chorioallantoic membrane (CAM assay) was evaluated.

## 2. Results

### 2.1. Extract Characterization

In order to identify the phytochemical profile of the Ej leaves aqueous extract, the total polyphenolic content and the antioxidant activity were determined, and an LC/MS analysis was also conducted. Our results indicated that the aqueous extract obtained from the Ej leaves had a total phenolic content of 605 mg GAE/100 g extract. The total antioxidant capacity determined with the CUPRAC (cupric reducing antioxidant capacity) method was 109 µmol Trolox equivalent per gram of dry matter (µmol TE/g).

LC/MS is a suitable analytical technique for separation, quantitative determination, and identification of phytochemicals, due to its sensitivity, speed, and structural elucidation capabilities [19]. The UHPLC method developed in this study was used to determine the polyphenolic compounds. The method enables simultaneous analysis of polyphenols using a single-pass column, with each LC–MS analysis completed within 14 min. The targeted UHPLC-MS/MS method monitored 15 polyphenolic standards. Of these, 8 were detected in the Ej aqueous leaf extract (7 quantified above the LOQ and quercetin identified qualitatively below the LOQ), while 4 were not found (Table 1).

The average recovery value was 101.03 ± 6.45%. Method precision was assessed on standard solutions for all monitored analytes. The mean intra-day RSD was 5.08%, with individual values ranging from 0.88% to 14.53% (gallic acid 3.68%, 3,5-/3,4-dihydroxybenzoic acid 3.51%, 2,4-dihydroxybenzoic acid 2.40%, chlorogenic acid 5.04%, caffeic acid 2.60%, p-coumaric/trans-p-coumaric acid 5.61%, (E/Z)-/trans-ferulic acid 4.40%, rutin 14.53%, resveratrol 12.73%, rosmarinic acid 0.88%, quercetin 2.95%, and genistein 2.58%). All values fall within the acceptable range for quantitative method validation (RSD < 15%), confirming the method is reliable for the compounds subsequently quantified in the extract (Table 1) [20].

The most abundant compound identified was chlorogenic acid with approximately 55,000 ng/mL. It is a phenylacrylate polyphenol produced in plants by the shikimic acid pathway during aerobic respiration [21].

The next identified compounds were rutin and the group of isomers 3,5-dihydroxybenzoic acid/3,4-dihydroxybenzoic acid, p-coumaric acid/trans-p-coumaric acid, 2,4-dihydroxybenzoic acid, caffeic acid, and ferulic acid (Table 1).

Only qualitatively, quercetin was identified at 5.90 min. Analyzing the signal-to-noise ratio (s/n) presented in Table 2, it was observed that 3,5-dihydroxybenzoic acid/3,4-dihydroxybenzoic acid, rutin, and chlorogenic acid have good values for the limit of quantification [22].

**Figure 1:**
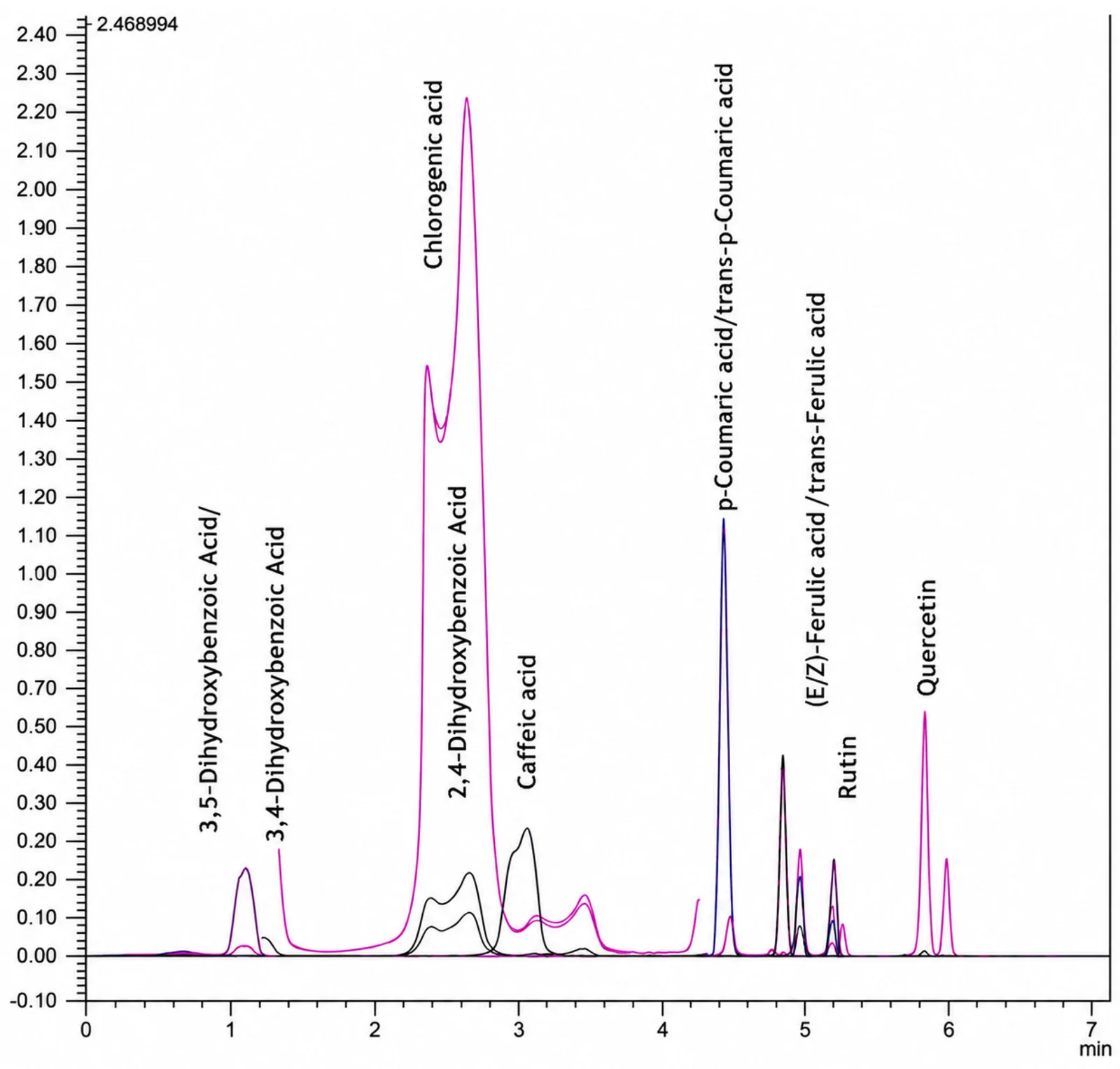
The total ion chromatogram for the Ej leaf extract.

The other compounds still have remarkable peaks at the expected retention times (Figure 1). Peak cleaning was attempted by dissolving the extract only in water or only in methanol, but the results did not significantly improve, with S/N for 2,4-dihydroxybenzoic acid being 0.11 in water and 0.33 in methanol, and for (E/Z)-ferulic acid 0.68 in water and 1.09 in methanol. In the case of caffeic and p-coumaric acids, S/N was not significantly modified, regardless of the extraction solvent.

### 2.2. Biological Assessment

#### 2.2.1. Ej Leaf Extract Induces a Dose-Dependent Reduction in Melanoma Cells Viability

**Figure 2:**
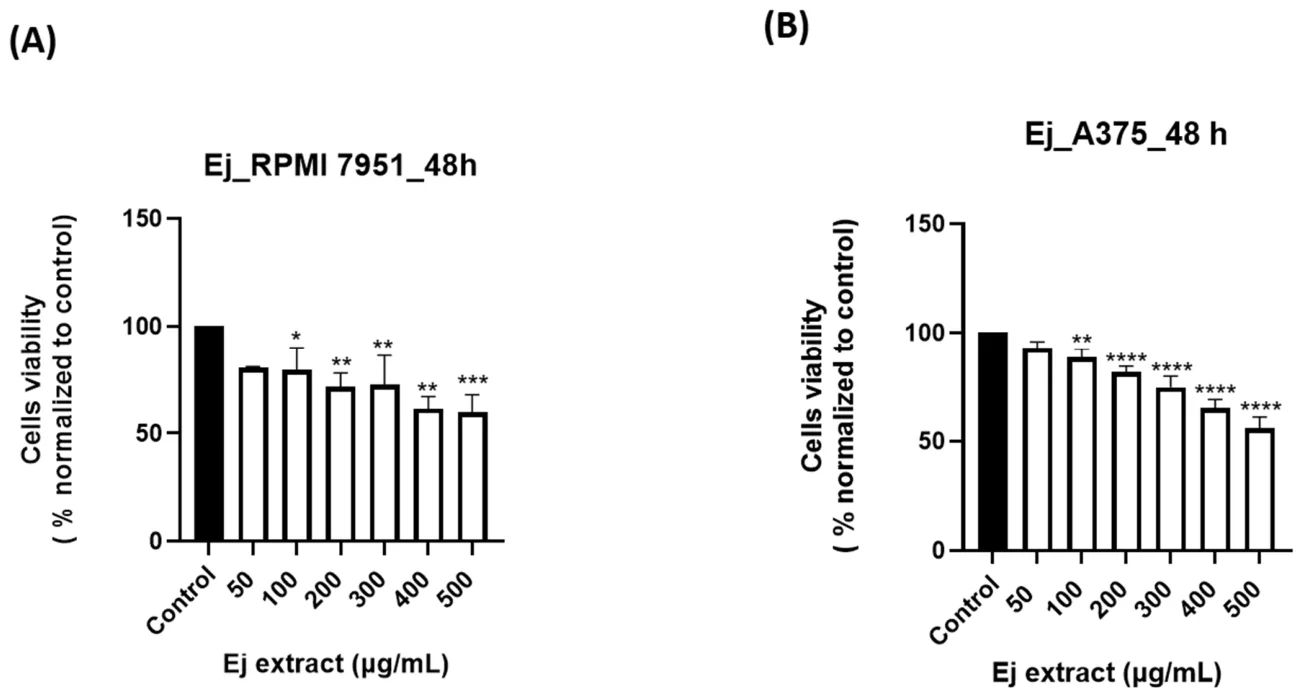
Cell viability percentages of RPMI 7951 (**A**) and A375 (**B**) cells after 48 h of treatment with Ej extract (50, 100, 200, 300, 400, and 500 µg/mL). The results are expressed as a percentage (%) relative to the untreated control group (negative control) and are presented as the mean ± standard deviation (SD) of three independent experiments, each performed in triplicate. Statistical differences between the control group and the treated groups were analyzed using the one-way ANOVA test, followed by the Dunnett’s post hoc test, and values of *p* < 0.05 were considered significant (*), * *p* < 0.05; ** *p* < 0.01; *** *p* < 0.001, **** *p* < 0.0001.

In the present study, the potential antitumor effect of Ej extract was evaluated in human primary (A375) and metastatic (RPMI 7951) melanoma cells. After a 48 h exposure of RPMI 7951 cells to increasing concentrations of Ej extract (50–500 µg/mL), the following viability percentages were calculated: 80.41%, 79.45%, 71.70%, 72.55%, 61.43%, and 59.87%, respectively (Figure 2A). Applying the same experimental conditions, for the A375 cells (Figure 2B), the following viability values were recorded at the same concentrations: 92.92%, 88.42%, 81.78%, 74.65%, 65.15%, and 56.18%. These data indicate a dose-dependent reduction in cell viability in both cell lines.

#### 2.2.2. Evaluation of the Cytotoxic Effect of Ej Leaf Extract on HaCaT Keratinocytes

**Figure 3:**
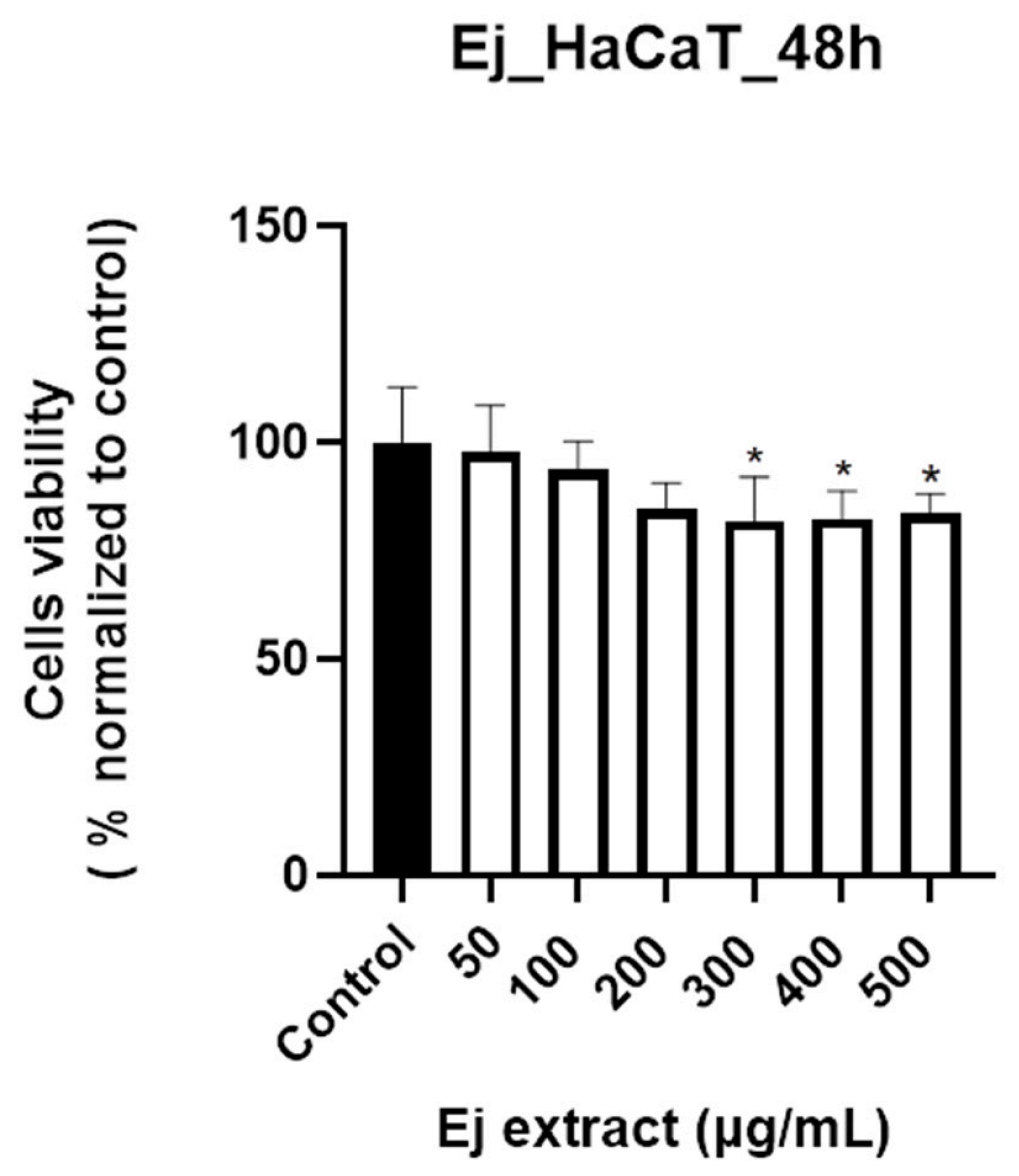
Cell viability percentages of HaCaT keratinocytes 48 h after exposure to Ej aqueous extract (50, 100, 200, 300, 400, and 500 µg/mL). Results are expressed as a percentage (%) relative to untreated control cells (negative control). Data are presented as mean ± standard deviation (SD) from three independent experiments performed in triplicate. Statistical differences between the control group and the treated groups were evaluated using one-way ANOVA followed by Dunnett’s post hoc test, and values of *p* < 0.05 were considered significant (*), * *p* < 0.05.

To assess whether the cytotoxic effects of the Ej extract were cell–type-dependent, its impact on non-tumoral HaCaT keratinocytes was also investigated. The potential in vitro cytotoxic effect of Ej extract on keratinocytes (HaCaT cells) was evaluated by employing the same experimental conditions as for human melanoma cells. The results indicated the following cell viability percentages: 97.86%, 93.70%, 87.80%, 87.19%, 86.26%, and 85.41%, respectively (Figure 3). Overall, a moderate reduction in cell viability was observed, with more pronounced effects occurring at the higher extract concentrations, still, according to the ISO 10993-5:2009 guidelines, the extract is not cytotoxic, since all concentrations maintained ≥70% cell viability. Thus, at the highest concentration tested (500 μg/mL), the extract caused a decrease in cell viability to 56.18% in the case of A375 cells (Figure 2B) and 59.87% in the case of RPMI-7951 cells (Figure 2A), while HaCaT keratinocytes maintained a viability of 85.41%. These results indicate a difference of approximately 29 and 26 percentage points, respectively. This preferential cytotoxic effect on tumor cells over non-tumor keratinocytes was observed over the entire concentration range and became more pronounced with increasing doses tested. Notably, the IC50 was not reached in either melanoma line under the tested conditions (50–500 µg/mL, 48 h); IC50 values are therefore estimated to exceed 500 µg/mL.

#### 2.2.3. Ej Leaf Extract Triggers Morphological Changes Dose-Dependently in Human Melanoma Cells

**Figure 4:**
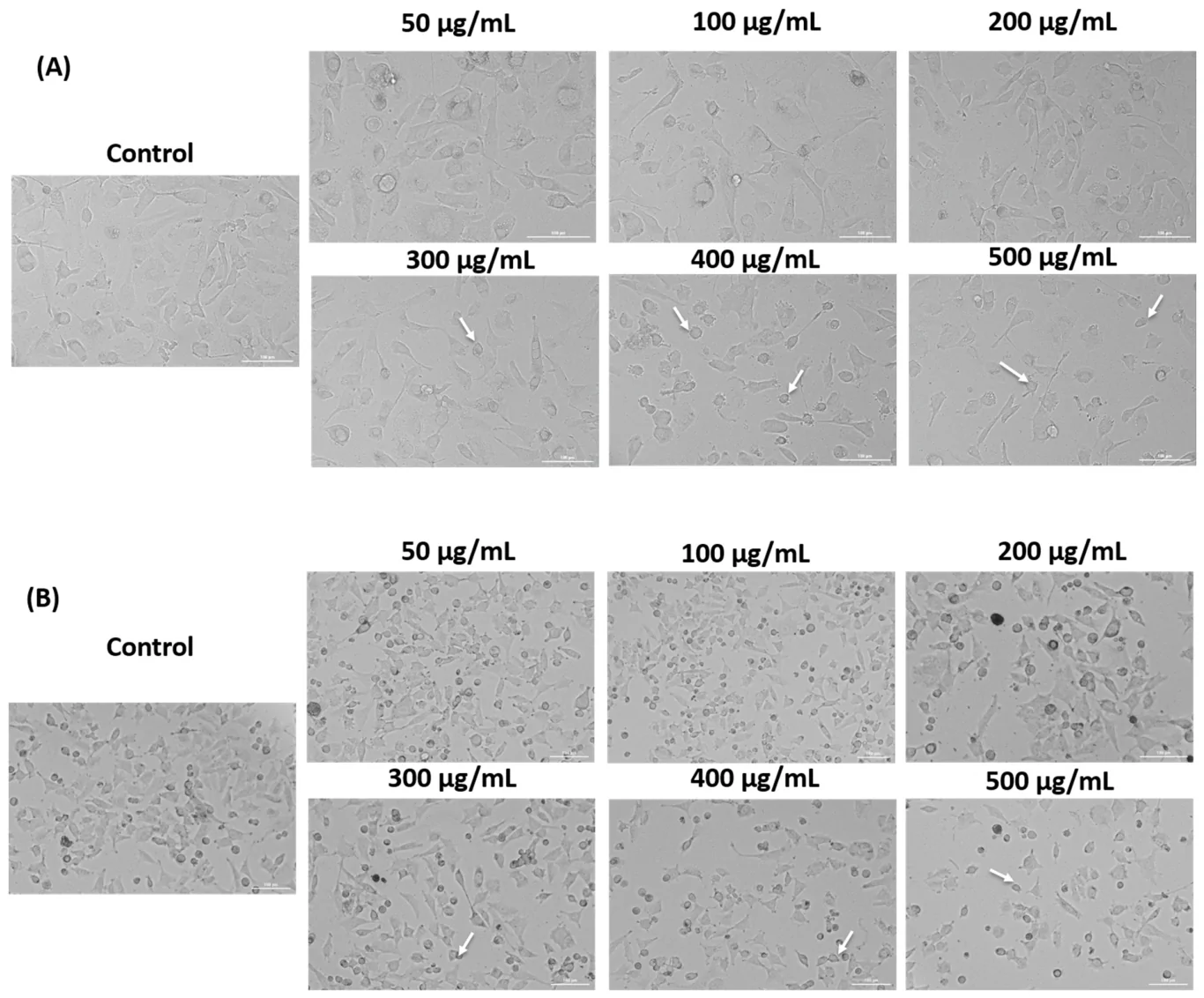
Representative bright-field microscopy images illustrating morphological alterations in (**A**) RPMI 7951 cells and (**B**) A375 melanoma cells after 48 h of exposure to the Ej extract at concentrations ranging from 50 to 500 µg/mL. Images were acquired at 20× magnification, and the scale bar represents 100 µm. The white arrows indicate cell shrinkage and rounding.

Furthermore, morphological evaluation of RPMI 7951 and A375 cells after 48 h of treatment with Ej extract (Figure 4) revealed visible, concentration-dependent changes in cell appearance and confluence. At low concentrations, both cell lines largely retained their typical morphology similar to the control cells (untreated cells), while a gradual reduction in cell confluence was observed with increasing concentrations. At higher doses, the treated cells showed reduced confluence and obvious morphological changes, including cell shrinkage and rounding, with partial detachment from the culture surface. These observations were consistent with the MTT test results (Figure 2), indicating a dose-dependent decrease in cell viability.

**Figure 5:**
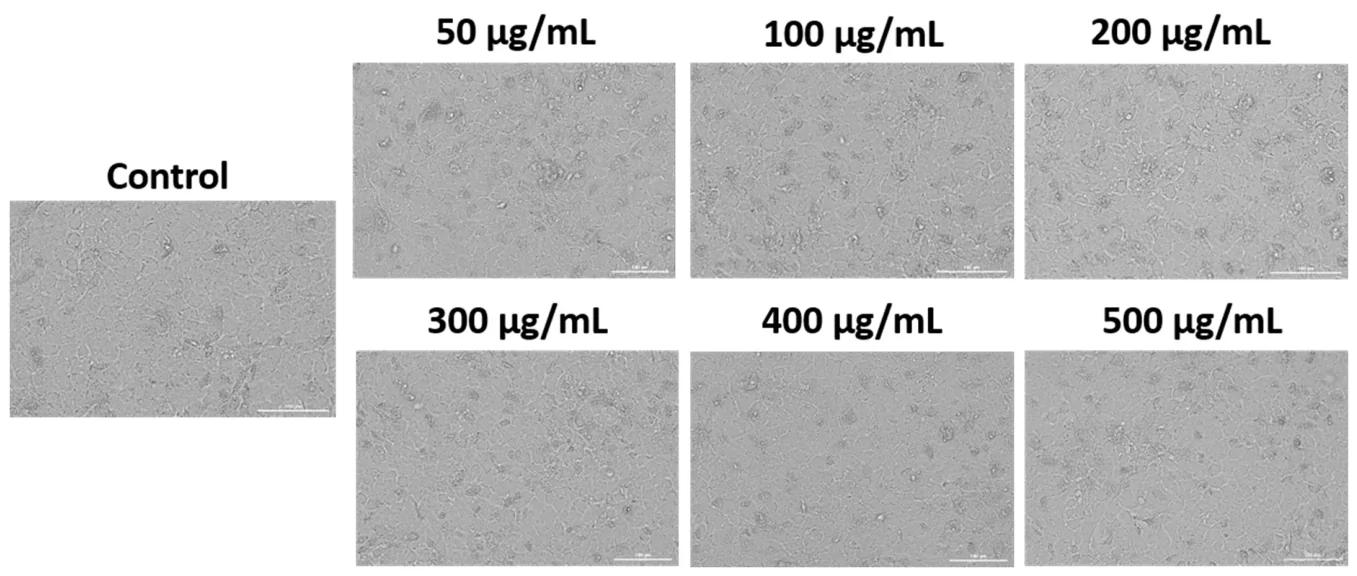
Representative bright-field microscopy images illustrating the morphological appearance of HaCaT keratinocytes after 48 h of exposure to the Ej extract at concentrations ranging from 50 to 500 µg/mL. The images are representative of each experimental condition and were acquired at 20× magnification; the scale bar represents 100 µm.

The influence of Ej extract on the morphology and confluence of HaCaT keratinocytes after 48 h of treatment is illustrated in Figure 5. At low concentrations, the morphological appearance was comparable to that of untreated cells. At the highest concentrations (400 and 500), only a slight reduction in cell confluence was observed, with the cellular structure remaining largely intact. These results are in line with those obtained in the MTT assay (Figure 3).

Given that both the MTT assay and the morphological assessment suggest that the Ej extract did not produce significant cytotoxic effects on HaCaT cells across the tested concentration range, no additional experiments were carried out on this cell line. Instead, subsequent analyses were focused on the melanoma cell lines.

#### 2.2.4. Ej Leaf Extract Impaired Lysosomal Function in Human Melanoma Cells

**Figure 6:**
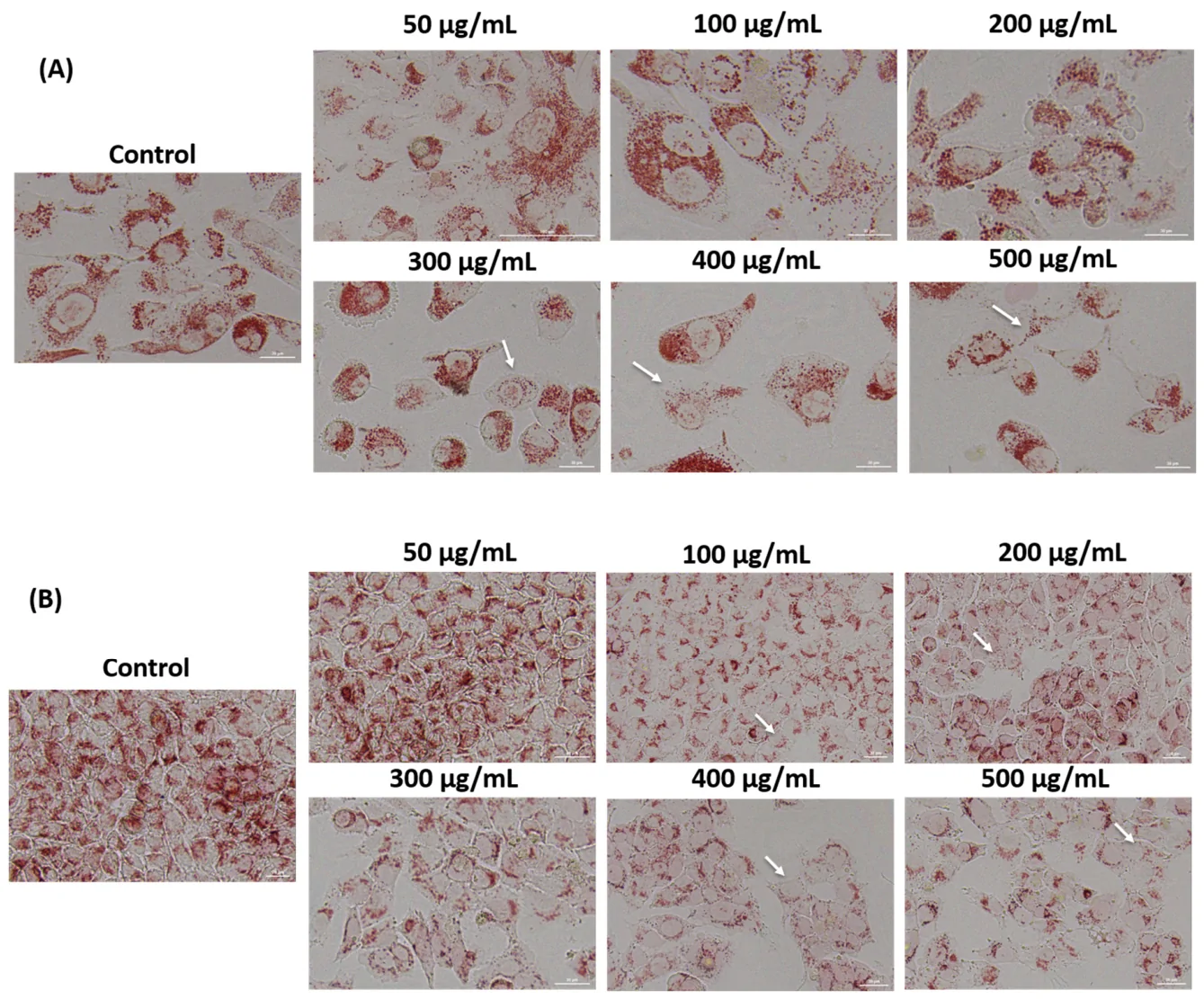
Representative images of Neutral Red staining in (**A**) RPMI 7951 and (**B**) A375 cells after 48 h of treatment with Ej extract at 50–500 μg/mL. The scale bar indicates 30 µm. The white arrows indicate cells with reduced retention capacity.

Since the MTT assay primarily reflects mitochondrial metabolic activity, the Neutral Red Uptake (NRU) assay was further performed to assess the potential involvement of lysosomal integrity in the cytotoxic effects induced by Ej extract after 48 h of treatment in melanoma cell lines. Microscopic examination revealed a progressive reduction in intracellular neutral red accumulation with increasing extract concentrations in both RPMI 7951 cells (Figure 6A) and A375 cells (Figure 6B). In both cell lines, cells in the control group showed uniform and intense internalization of the dye, indicating the integrity of the lysosomes. In the case of cells treated with the extract, a gradual reduction in the internalization of the dye was observed, particularly from a concentration of 300 µg/mL onwards. At concentrations of 400 and 500 µg/mL, the reduction in dye uptake was most pronounced, indicating compromised lysosomal integrity following treatment.

**Figure 7:**
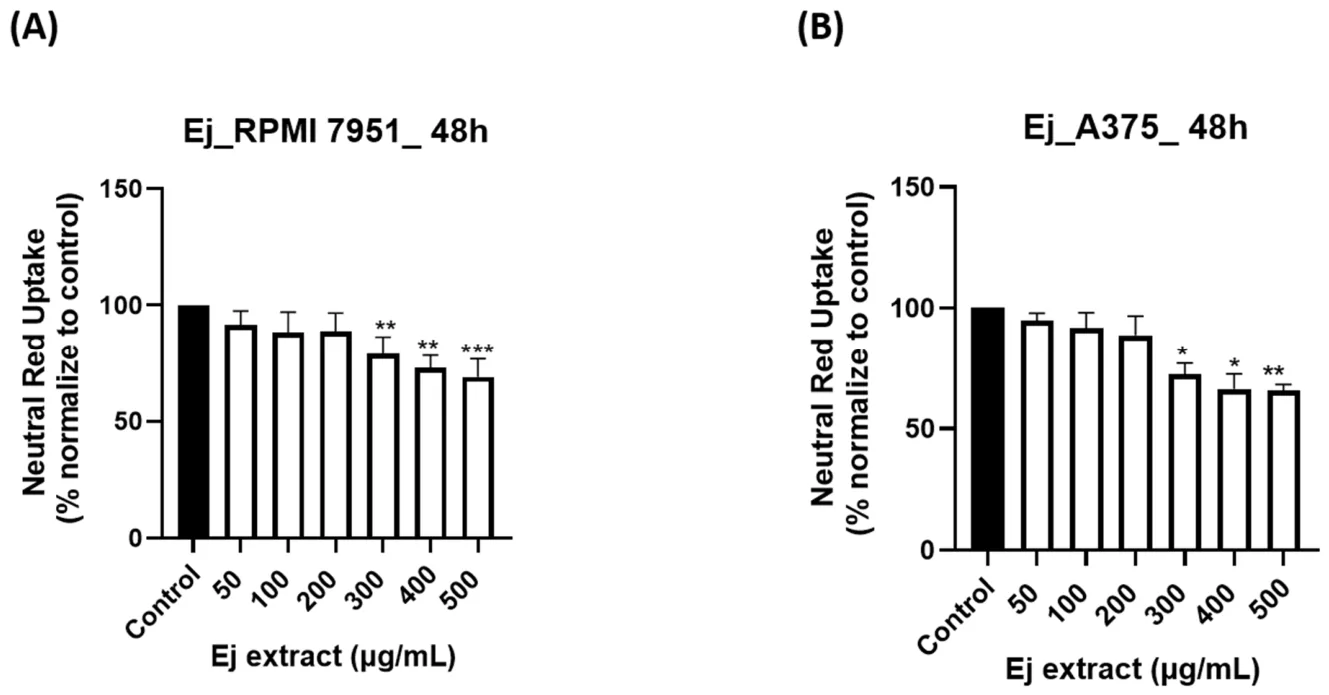
Graphical representation of Neutral Red dye absorption in (**A**) RPMI 7951 cells and (**B**) A375 cells after treatment with Ej extract. Cells were incubated for 48 h with Ej extract (50–500 µg/mL). The values are expressed as a percentage relative to untreated control cells (negative control) and are presented as mean ± standard deviation (SD), obtained from three independent experiments, each performed in triplicate. The analysis of statistical differences between the control group and the treated samples was performed using the one-way ANOVA test, followed by the Dunnett’s post hoc test for multiple comparisons. Statistical significance levels are indicated by the symbols * (*p* < 0.05), ** (*p* < 0.01), *** (*p* < 0.001). The values corresponding to the control are represented graphically in black.

Quantitative analysis (Figure 7) indicated the following NRU (Neutral Red Uptake) results for RPMI 7951 cells after 48 h of treatment with the Ej extract (50, 100, 200, 300, 400, and 500 µg/mL): 91.65%, 88.22%, 88.86%, 79.12%, 72.92%, and 68.95%, respectively. While for A375 cells, the following results were obtained for the same tested concentrations: 94.89%, 91.55%, 88.86%, 72.45%, 66.25%, and 65.61%, respectively.

#### 2.2.5. Ej Leaf Extract Stimulates ROS Production in a Dose-Dependent Manner in Human Melanoma Cells

**Figure 8:**
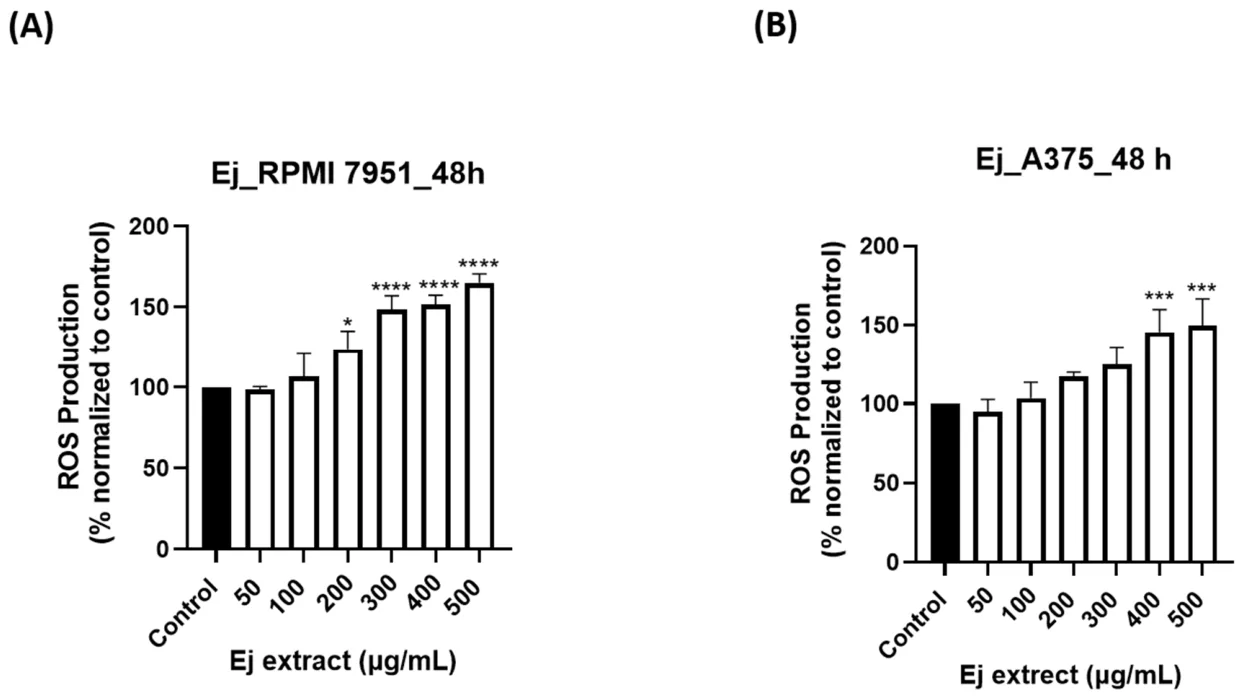
Graphical representation of the effects induced by Ej leaf extract (50, 100, 200, 300, 400, and 500 µg/mL) on intracellular ROS production in melanoma cells after 48 h of treatment: (**A**) RPMI 7951 cells and (**B**) A375 cells. The results are expressed as a percentage (%) relative to the untreated control group (negative control) and are presented as the mean ± standard deviation (SD) of three independent experiments, each performed in triplicate. Statistical differences between the control group and the treated groups were analyzed using the one-way ANOVA test, followed by the Dunnett’s post hoc test, and values of *p* < 0.05 were considered significant (*), * *p* < 0.05, *** *p* < 0.001, **** *p* < 0.0001.

In order to explore the mechanism of cytotoxicity, the evaluation of the Ej extract was further continued by assessing its impact on ROS production in both melanoma cell lines. For the RPMI 7951 cells (Figure 8A), the results obtained after 48 h of treatment indicated the following ROS production levels: at concentrations of 50, 100, 200, 300, 400, and 500 µg/mL, the recorded ROS levels were 98.35%, 106.94%, 123.90%, 148.37%, 151.89%, and 165.06%, respectively. Regarding the intracellular ROS production in A375 cells (Figure 8B), under the same conditions, the results showed a similar trend as for RPMI 7951 cells: 95.02%, 103.61%, 117.36%, 125.04%, 145.56%, and 149.09%. These results show an increase in ROS production correlated with increasing concentrations of Ej extract and cytotoxicity, respectively.

#### 2.2.6. Ej Leaf Extract Induces Alterations of Mitochondrial Membrane Potential in Human Melanoma Cells

**Figure 9:**
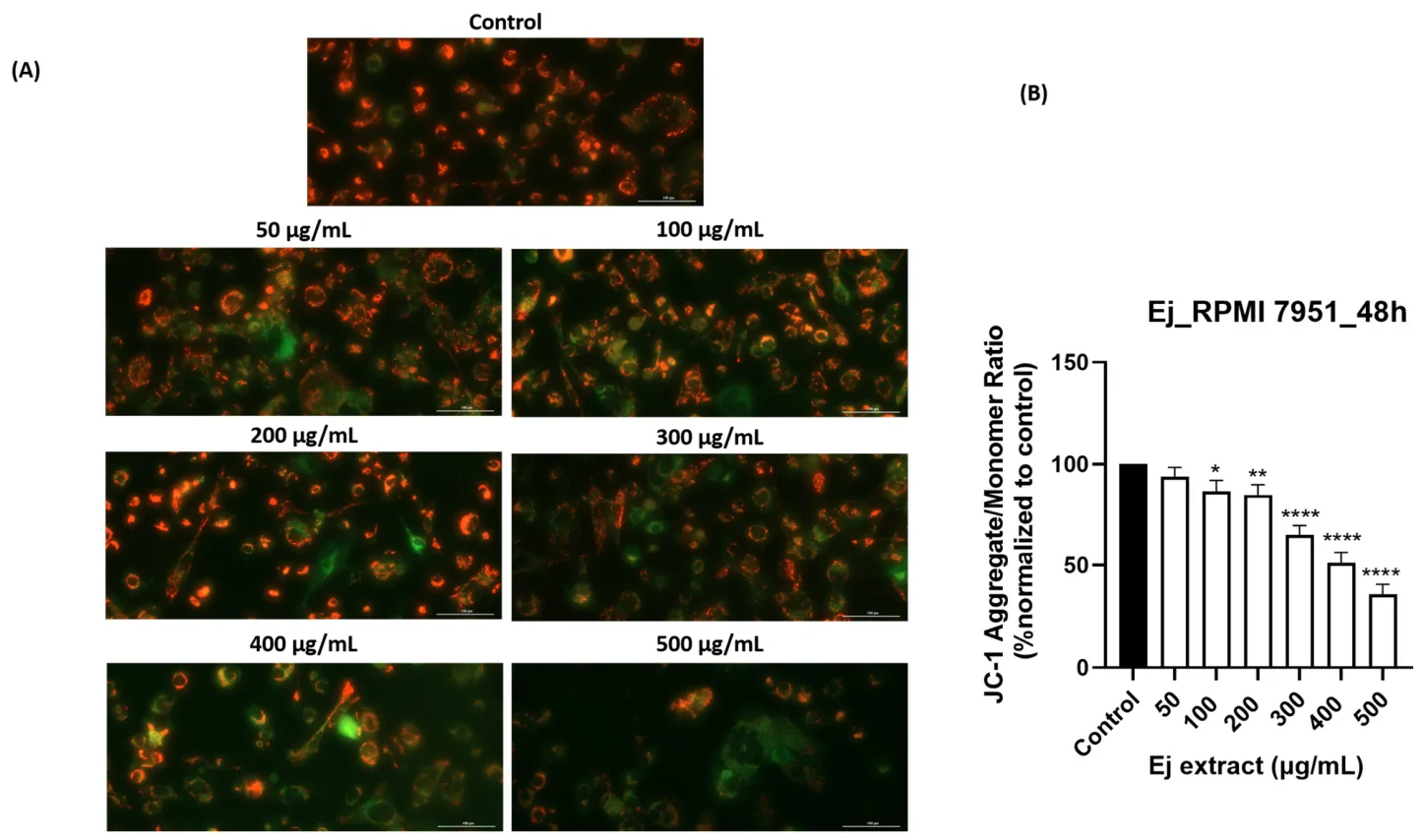
(**A**) The effect of Ej extract (50–500 µg/mL) on the mitochondrial membrane potential of RPMI 7951 cells. The scale bar indicates 100 µm. (**B**) Graphical representation of JC-1 aggregate/monomer ratio expressed as a percentage normalized to untreated cells (negative control). Results are presented as mean values ± standard deviation from three independent experiments, each performed in triplicate. To evaluate statistical differences between the control and treatment groups, a one-way ANOVA test was conducted, followed by Dunnett’s multiple comparison post hoc test. “*” indicates statistical significance (* *p* < 0.05, ** *p* < 0.01, **** *p* < 0.0001).

As illustrated in Figure 9A,B, the JC-1 assay revealed dose-dependent alterations in mitochondrial membrane potential following 48 h of exposure to the Ej extract in RPMI 7951 cells. A progressive, concentration-dependent reduction in mitochondrial membrane potential was observed, characterized by an increase in green fluorescence and a concomitant decrease in red fluorescence. Accordingly, the JC-1 aggregate/monomer ratio declined to 93.99%, 86.56%, 84.89%, 65.35%, 51.03%, and 35.69% at Ej extract concentrations of 50, 100, 200, 300, 400, and 500 µg/mL, respectively.

**Figure 10:**
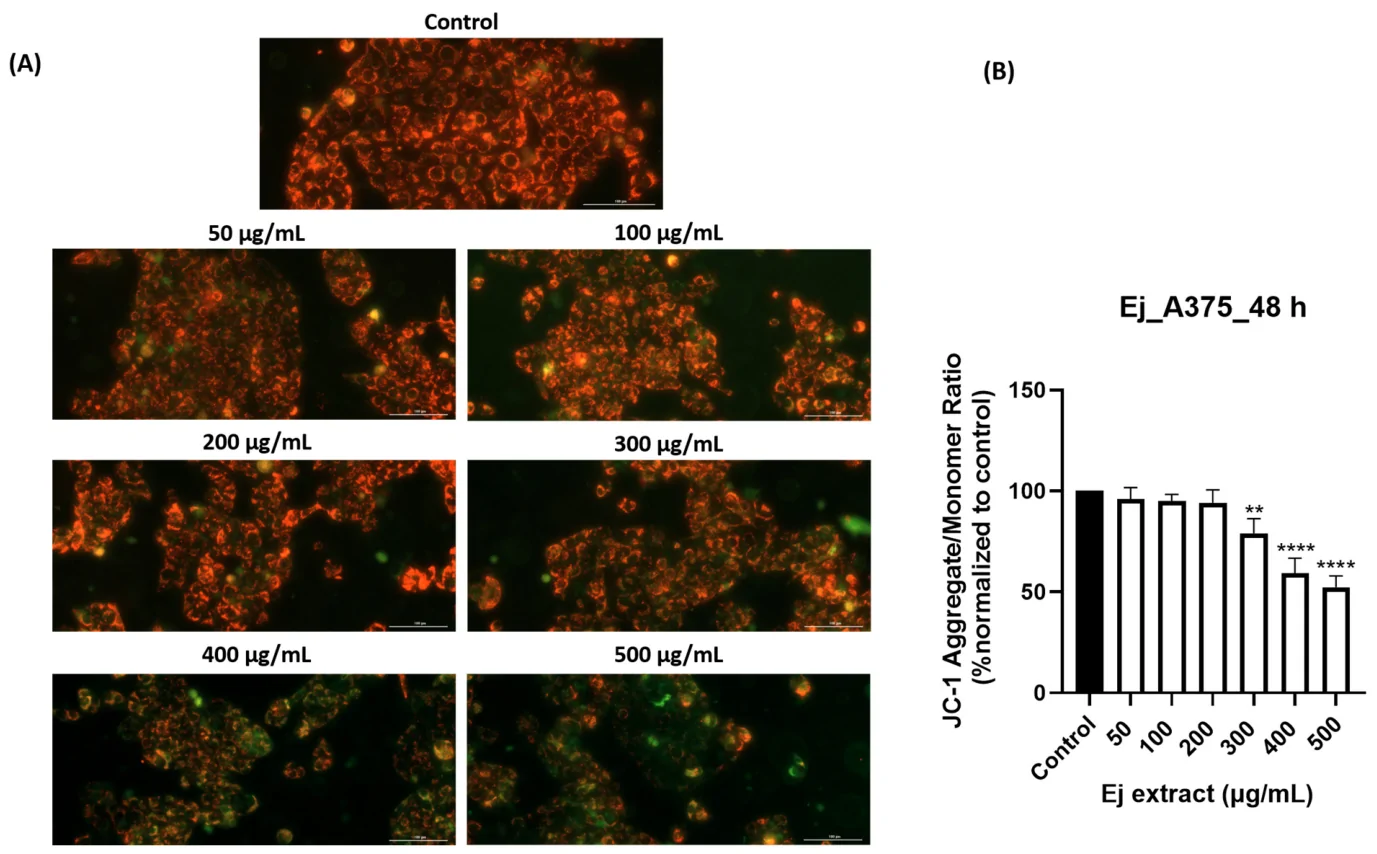
(**A**) The effect of Ej extract (50–500 µg/mL) on the mitochondrial membrane potential of A375 cells. The scale bar indicates 100 µm. (**B**) Graphical representation of JC-1 aggregate/monomer ratio expressed as a percentage normalized to untreated cells (negative control). Results are presented as mean values ± standard deviation obtained from three independent experiments, each performed in triplicate. To evaluate statistical differences between the control and treatment groups, a one-way ANOVA test was conducted, followed by Dunnett’s multiple comparison post hoc test. “*” indicates statistical significance (** *p* < 0.01; **** *p* < 0.0001).

The results of the JC-1 assay on A375 cells following treatment with the Ej extract (Figure 10A) showed that at a concentration of 200 µg/mL, the reduction in mitochondrial membrane potential was very slight, with red fluorescence remaining predominant at a level comparable to the control. Starting from the concentration of 300 µg/mL, a more pronounced decrease in red fluorescence was observed, indicating a reduction in mitochondrial membrane potential, with the most evident effect seen at concentrations of 400 and 500 µg/mL. Moreover, the quantitative analysis (Figure 10B) of the treatment’s impact on mitochondrial membrane potential, assessed by determining the JC-1 aggregate/monomer ratio, indicated the following results: 95.89%, 94.88%, 94.19%, 79.12%, 59.26%, and 52.29% for the concentrations of 50, 100, 200, 300, 400, and 500 µg/mL, respectively.

#### 2.2.7. Ej Leaf Extract Triggers Changes in Mitochondria and Nuclei Appearance in Melanoma Cells

**Figure 11:**
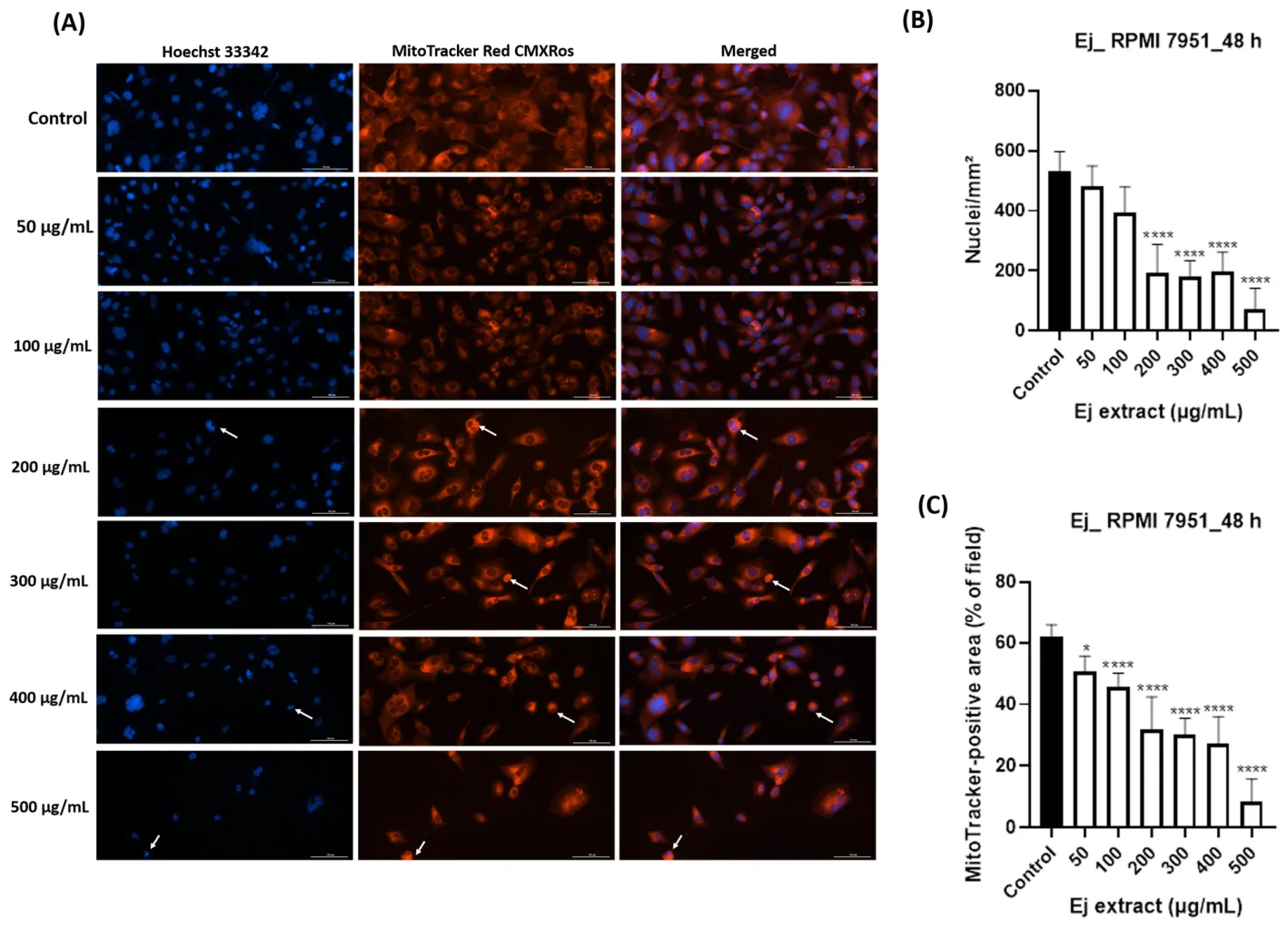
Effect of Ej extract on nuclear and mitochondrial morphology in the RPMI 7951 melanoma cell line after 48 h of treatment. (**A**) Representative fluorescent images of cells, highlighting nuclei (Hoechst 33342 blue staining) and mitochondria (MitoTracker Red CMXRos red staining), as well as the corresponding merged images. Images were taken at 20× magnification; scale bar: 100 µm. White arrows indicate significant qualitative changes observed, such as rounding and decreased spreading. (**B**) Nuclear density, expressed as the number of nuclei per mm^2^ of the analyzed field. (**C**) MitoTracker-positive area, expressed as the percentage of the analyzed field occupied by mitochondrial signal. In (**B**,**C**), data are represented as mean ± SD of four non-overlapping subfields of the representative image analyzed for each treatment condition. Statistical differences between the control and treated groups were assessed by one-way ANOVA followed by Dunnett’s multiple comparison test (* *p* < 0.05; **** *p* < 0.0001).

By applying the fluorescence microscopy method, RPMI 7951 cells stained with Hoechst 33342 and MitoTracker Red CMXRos, at the time interval of 48 h of treatment, a series of dose-dependent changes were revealed (Figure 11A). Cells exposed to the lowest concentrations (50 and 100 µg/mL) maintained a nuclear and mitochondrial appearance comparable to that of the untreated control group. Starting with the concentration of 200 µg/mL, the most obvious change was the progressive loss of cellular confluence, associated with the uneven distribution of the mitochondrial signal. The highest concentrations, of 400 and 500 µg/mL, led to a decrease in adherent cells, and the residual cells were characterized by a reduced and irregular mitochondrial network. Quantitative analysis confirms the aforementioned observations, nuclear density (Figure 11B) decreased from 530.14 nuclei/mm^2^ in untreated controls to 191.93, 181.56, 197.12 and 72.62 nuclei/mm^2^ at 200, 300, 400 and 500 µg/mL respectively (*p* < 0.0001 for all four), while no significant change was detected at 50 and 100 µg/mL. Also, the MitoTracker area (Figure 11C) showed the same pattern, decreasing from 62.03% of the analyzed field in the control group to 31.93% at 200 µg/mL and to 8.38% at 500 µg/mL (*p* < 0.0001), with a significant reduction already evident at 100 µg/mL (45.73%, *p* < 0.05).

**Figure 12:**
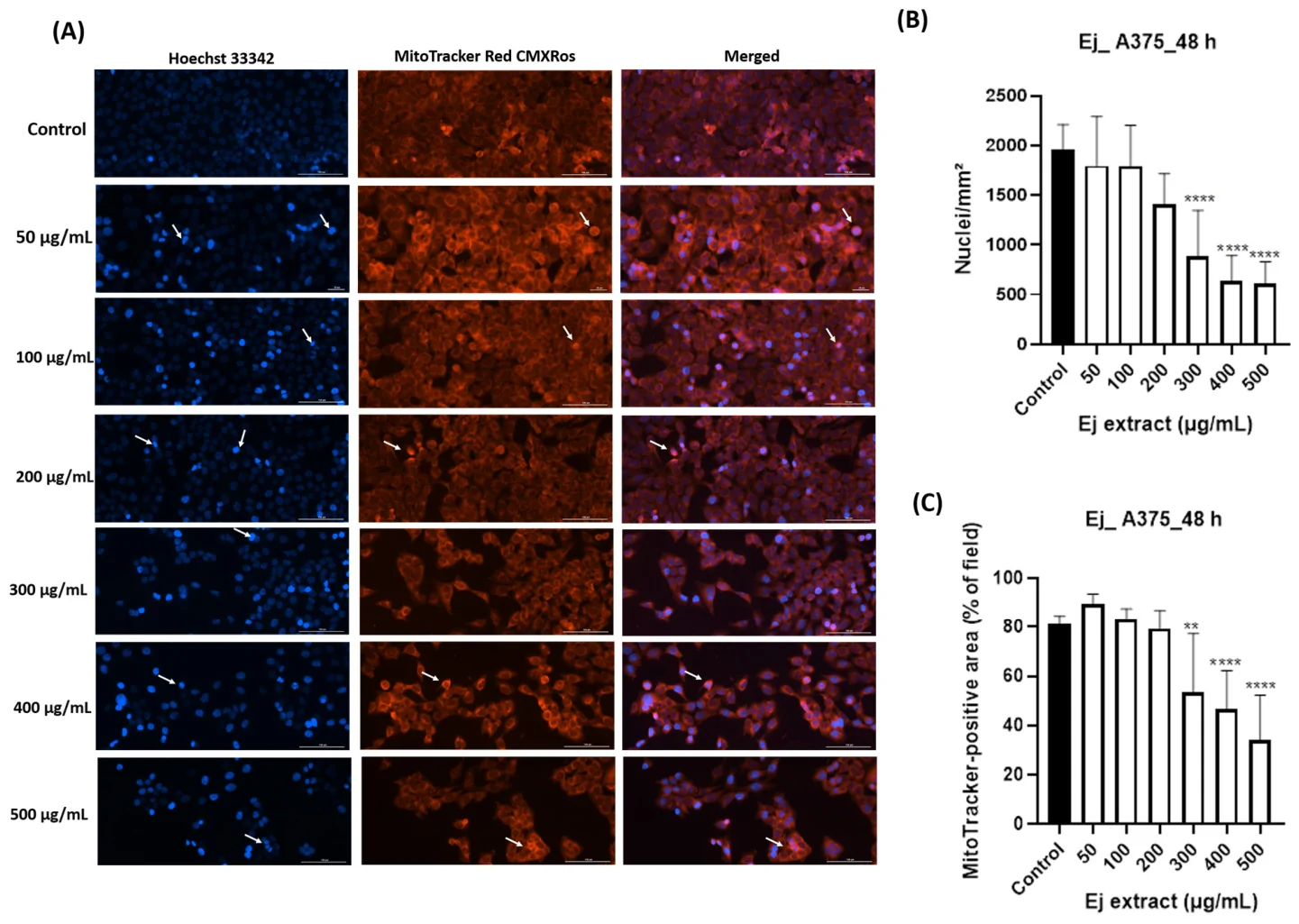
Effect of Ej extract on nuclear and mitochondrial morphology in the A375 melanoma cell line after 48 h of treatment. (**A**) Representative fluorescent images of cells, highlighting nuclei (Hoechst 33342 blue staining) and mitochondria (MitoTracker Red CMXRos red staining), as well as the corresponding merged images. Images were taken at 20× magnification; scale bar, 100 µm. White arrows indicate significant qualitative changes observed, such as rounding and decreased spreading. (**B**) Nuclear density, expressed as the number of nuclei per mm^2^ of the analyzed field. (**C**) MitoTracker-positive area, expressed as the percentage of the analyzed field occupied by mitochondrial signal. In (**B**,**C**), data are represented as mean ± SD of four non-overlapping subfields of the representative image analyzed for each treatment condition. Statistical differences between the control and treated groups were assessed by one-way ANOVA followed by Dunnett’s multiple comparison test (** *p* < 0.01; **** *p* < 0.0001).

Similar changes were also observed in A375 cells (Figure 12A). Low concentrations, up to 200 µg/mL, did not induce relevant changes in nuclear and mitochondrial morphology. At concentrations starting from 300 µg/mL, a significant decrease in the number of adherent cells was revealed, together with smaller nuclei that were intensely stained, changing the intensity of a mitochondrial signal limited to a smaller cell surface. Quantification of the observed changes indicated that significant effects occurred at higher concentrations compared to RPMI 7951 cells. Thus, nuclear density (Figure 12B) remained unchanged up to a concentration of 200 µg/mL with significant decreases from 1956.85 nuclei/mm^2^ in the control group to 888.80, 639.30 and 608.12 nuclei/mm^2^ at 300, 400 and 500 µg/mL. Regarding the MitoTracker-positive area (Figure 12C), a decrease was observed from 81.13% to 53.49%, 46.90%, and 34.08% at the same concentrations. These results are consistent with those previously obtained in cell viability assays (Figure 2B) and evaluation of mitochondrial membrane potential by the JC-1 assay (Figure 10).

#### 2.2.8. Irritant Potential of Ej Extract on the CAM (Chick Chorioallantoic Membrane)

**Figure 13:**
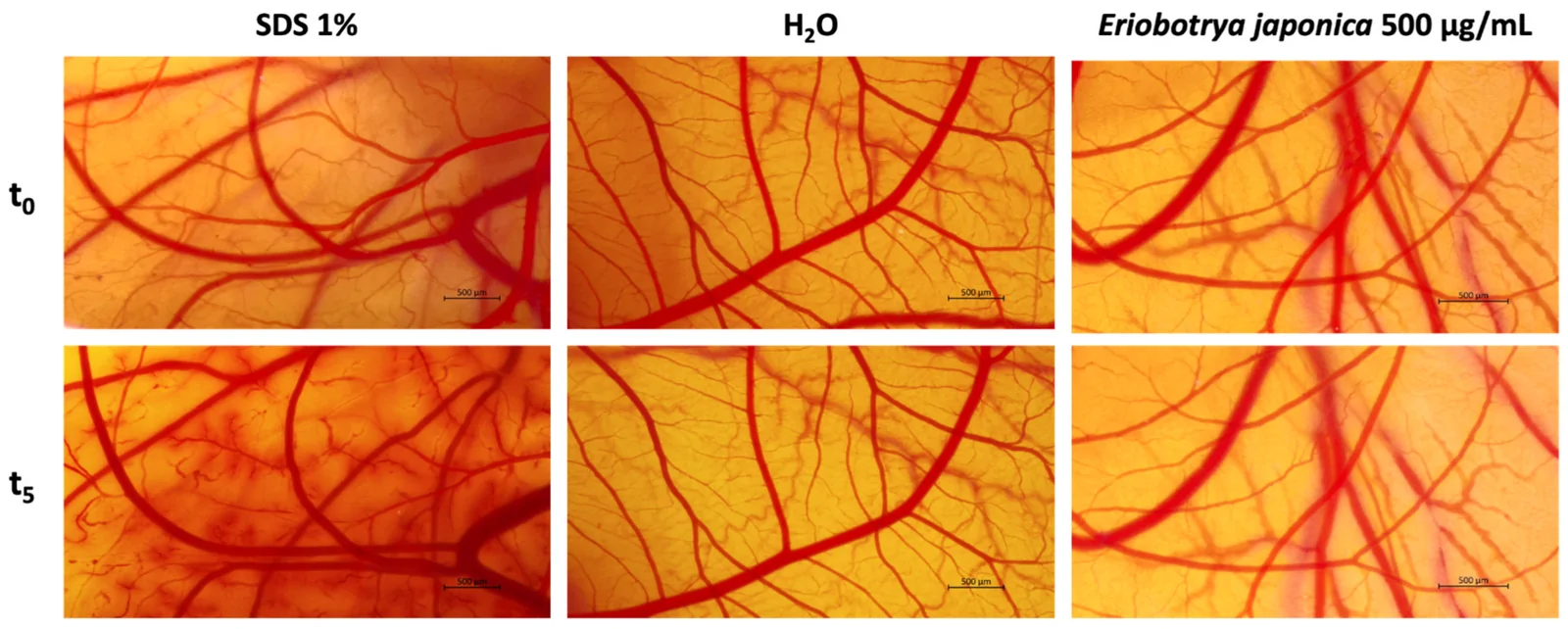
HET-CAM assay. Stereomicroscopic images revealing the chorioallantoic membrane (CAM) prior to the application of Ej extract (500 μg/mL), alongside positive control (sodium dodecyl sulfate) and negative control (distilled water). Images were captured 5 min post-application, emphasizing the morphological changes observed in the CAM. The scale bar represents a measurement of 500 μm.

The Hen’s egg test on chorioallantoic membrane (HET-CAM) assay was performed to evaluate the acute irritant potential of the Ej leaf extract on the highly vascularized CAM model (Figure 13). The concentration of 500 μg/mL was chosen based on preliminary results obtained in vitro. Assessment was performed by tracking irritation reactions in the chorioallantoic membrane of chicken embryos, as indicated by markers including hemorrhage, coagulation, or lysis. The irritability score (IS) was then calculated, which facilitated the classification of the tested extract in accordance with the criteria established by Luepke [23]. This classification divides substances into four groups: non-irritating (IS = 0–0.9), mildly irritating (IS = 1–4.9), moderately irritating (IS = 5–8.9), and strongly irritating (IS = 9–21). At a concentration of 500 µg/mL, the Ej extract caused no hemorrhage, vascular lysis, or coagulation during the 300 s period of observation. An IS of 0.07 was obtained, which is equal to that of the reference negative control (distilled water), which classifies the extract as non-irritating on the Luepke scale. In comparison, the positive control (1% SDS) caused rapidly occurring vascular alterations, resulting in an IS of 14.3 ± 2.0, indicating a strong irritant response (Table 3). These observations indicate that the Ej extract showed no acute vascular irritant effects on CAM at 500 µg/mL. It is noteworthy that this concentration ranged in the interval of biological activity observed in vitro, where the extract showed a cytotoxic effect in melanoma cells, while preserving a more moderate effect on HaCaT keratinocytes.

## 3. Discussion

In the present study, we explored the biological effects of loquat, principally the in vitro antitumoral (antimelanoma) potential, and for this purpose, a leaf aqueous extract was prepared and physicochemical and toxicologically analyzed. The main findings of the study consisted of: (i) the obtention of an Ej aqueous extract rich in chlorogenic acid, with an elevated total phenolic content (605 mg GAE/100 g extract) and a high antioxidant potential (109 µmol TE/g); (ii) the Ej extract reduced melanoma cell (RPMI 7951 and A375) viability in a concentration-dependent manner, although it exerted only a mild effect on non-tumor keratinocytes, HaCaT, indicating a preferential action against melanoma cells in relation to this type of non-tumor skin cells; and (iii) the cytotoxicity in melanoma cells was characterized by a decrease in cell viability, apoptotic-like specific changes in melanoma cell shape, impaired lysosomal integrity, increased ROS production (dose-dependent), loss of mitochondrial membrane potential, and mitochondrial network and nuclear alterations. In addition, based on the Luepke criteria [23], the Ej extract showed no irritant potential when tested at the highest concentration—500 μg/mL—on chick chorioallantoic membrane.

Our findings concerning the total phenolic content determined in the analyzed Ej extract indicated a value of 605 mg GAE/100 g extract, suggesting a relatively high abundance of phenolic compounds. Phenolic compounds, mainly polyphenols, are significant constituents of medicinal plants, exhibiting a range of beneficial biological effects, such as cardioprotective, antiviral, antimicrobial, and anticancer effects [24]. The total polyphenolic content value obtained is considerably higher than those previously reported for Ej fruits and related preparations. For instance, Zhang et al. reported total phenolic contents ranging from 30.58 to 43.70 mg GAE/g DW for the peel and from 9.90 to 13.73 mg GAE/g DW for the pulp [25], while Pawłowska et al. described significantly lower values, around 6.05 mg GAE/100 g [13]. Similarly, Xu et al. reported values ranging from 24 to 57 mg GAE/100 g among different varieties of Ej fruits [26]. In addition, previous studies have shown that the harvesting season and the environmental conditions can contribute to variations in the content of bioactive compounds in the leaves of Ej, including the content of phenolic compounds and flavonoids [27,28]. Such variations can be due to several reasons, including the part of the plant used (leaves and/or fruits), extraction technique, solvent polarity, and the conditions of growth, all of which greatly affect the amount of total phenolic content. In previous studies, a clear relationship was established between the total phenolic content and the phenolic content. It is especially true for leaves, as the latter tend to contain higher concentrations of polyphenols than the fruits [29]. Furthermore, in previous studies, a clear relationship was established between the total phenolic content and the antiproliferative activity, thus indicating that phenolic compounds are the major components responsible for cytotoxic activity [30]. In this context, we hypothesized that the relatively high phenolic content of the Ej extract may contribute to the antimelanoma potential shown in this study.

By conducting the LC-MS analysis, we quantified seven of the fifteen monitored compounds in the extract, as follows: chlorogenic acid, rutin, 3,5-dihydroxybenzoic acid/3,4-dihydroxybenzoic acid, p-coumaric ac-id/trans-p-coumaric acid, 2,4-dihydroxybenzoic acid, caffeic acid, and (E/Z)-ferulic acid/trans-ferulic acid, supporting the hypothesis that the observed biological activities may be closely linked to its phytochemical composition. Chlorogenic acid was the most abundant compound identified in the extract, followed by rutin. In a previous study, when the phytochemical content of an aqueous extract obtained from loquat leaves cultivated in Morocco was analyzed by the HPLC method, ten compounds were identified, rutin being among the more abundant flavonoids (7.55 ± 0.12 mg/g content), whereas chlorogenic acid had a 5.94 ± 0.08 mg/g content. The value of chlorogenic acid was comparable with our own results [18]. Still, the differences in the phytochemical profile and the abundance of individual compounds could be associated with the different method of extraction used and also the different environmental conditions (Romania versus Morocco). In another study, a leaf methanol extract obtained from loquat cultivated in Veracruz, Mexico, was analyzed by UPLC-QqQ-MS analysis, and a number of 22 compounds were revealed, mainly flavonoids and phenolic acids [31], results that support our data.

Most of the compounds identified within the Ej extract are well-known for their complex biological activities, such as hepato- and reno-protective, antioxidant, antibacterial, antitumor, anti-inflammatory, neuroprotective, antidiabetic, and dermoprotective [32,33,34,35,36,37,38,39,40,41,42].

Hence, considering the phenolic profile identified by LC-MS analysis and the well-documented antioxidant properties of polyphenolic compounds, the study was further directed toward the evaluation of the total antioxidant capacity of the extract. Total antioxidant potential measured using the CUPRAC technique was found to be 109 µmol Trolox equivalents/g dry weight, implying the presence of higher antioxidant potential. This finding is in line with the high phenolic concentration of the extract because polyphenols are known to play a major role in antioxidants as electron donors [43]. Comparable antioxidant properties have been reported for Ej, although the values vary considerably depending on factors such as the plant part, extraction conditions, and analytical method used. In general, leaf extracts tend to show higher antioxidant activity than fruits, which could explain the relatively high value obtained in the present study [44].

Recently, there has been a significant rise in interest in using natural compounds as anticancer agents, mainly because of their chemical diversity and ability to modulate multiple cellular processes with reduced impact on normal cells [45]. A key way many plant-derived compounds fight cancer is by triggering programmed cell death, a carefully regulated process that removes damaged or abnormal cells. When apoptosis is dysregulated, it often contributes to cancer development and progression, as cancer cells can bypass this process and grow unchecked. For this reason, therapeutic strategies aimed at reactivating cancer cells’ sensitivity to apoptosis are a major focus of current oncology research [46].

Since the phytochemicals identified in the Ej leaf extract (chlorogenic acid, rutin, caffeic acid, etc.) modulate multiple molecular targets related to carcinogenesis, as cell death (apoptosis), ROS production/inhibition, cell cycle and inflammation [47,48,49,50], the study continued with an evaluation of the cytotoxic potential of the Ej leaf extract in vitro, using two human melanoma cell lines (RPMI 7951 and A375), as well as non-tumor human keratinocytes (HaCaT). Untreated cells were used as the reference control because the final DMSO concentration in all treatment groups did not exceed 0.5%, a concentration generally considered to have minimal effects on mammalian cell viability and morphology. Therefore, the observed effects were attributed primarily to the plant extract. In both melanoma cell lines, the Ej extract reduced viability in a concentration dependent manner, with a slightly greater reduction in cell viability observed in A375 cells. These observations indicate cell-type-dependent variability, frequently reported for natural compounds [51].

So far, most of the published articles related to antimelanoma activity of Ej extracts (flower and leaves) employed as biological model the B16 murine melanoma cells [52,53]; therefore, the studies on human melanoma cells are rather scarce, our results representing an element of novelty in this regard. However, the antitumor potential of Ej extracts was proved against other tumor cell lines, including breast adenocarcinoma (MCF-7, MDA-MB-231), colon adenocarcinoma (Caco-2 and HT-29), glioblastoma (U87MG) [13,14], liver (HepG2) and lung (A549) cancers [12].

Regarding the impact of Ej extract on HaCaT cells viability, a decrease in viability percentage was observed for the higher concentrations tested (400 and 500 µg/mL) at 86.26% and 85.41%, respectively (Figure 3). However, according to the ISO 10993-5:2009 guidelines, the extract is not cytotoxic, since all concentrations did not decrease the viability percentage below 70%. Overall, these results suggest that the Ej extract exerts a dose-dependent cytotoxic effect on melanoma cell lines, while having a moderate impact on normal cells. This preferential effect toward tumor cells, relative to non-tumoral keratinocytes, was evident across the entire concentration range: at 500 µg/mL, melanoma viability decreased to 56.18% (A375) and 59.87% (RPMI-7951), whereas HaCaT keratinocytes retained 85.41% viability—a difference of ~29 and ~26 percentage points, respectively. It should be noted, however, that the IC50 was not reached in either melanoma line within the tested range (50–500 µg/mL), indicating IC50 values above 500 µg/mL and a moderate rather than potent cytotoxic potency. In support of our findings, previous studies on Ej leaf extracts have reported a minimal cytotoxic effect on keratinocyte models at moderate concentrations, with cell viability remaining close to control levels under similar exposure conditions [53]. It has been demonstrated that Ej extracts provide protective and antioxidant actions in stressed keratinocytes. This evidence shows that the biological activity of these extracts on healthy skin cells is associated with modulation of homeostatic mechanisms rather than with cytotoxic effects [54].

In addition to the decrease in viability of melanoma cells in the MTT assay, morphological changes were also detected in bright-field microscope images after 48 h of treatment with Ej extracts (Figure 4). These morphological alterations were more evident at higher concentrations and were characterized by decreased cell confluence, shrinking and rounding of cells, as well as incomplete attachment of the cells to the substrate—phenomena typical of the apoptotic process. The above evidence corroborates the data of the MTT assay and demonstrates the dose-dependent effect of Ej extracts on melanoma cell viability and indicates cytotoxic activity in these cells.

Since the MTT assay depends on mitochondrial metabolism, a reduction in viability may suggest mitochondrial dysfunction. For this reason, additional experiments were conducted to reveal the mechanisms involved, such as lysosomal integrity, levels of oxidative stress, and mitochondrial membrane potential.

Furthermore, the cytotoxicity test was performed using another method, the Neutral Red Uptake (NRU) test, which determines cell viability by measuring the integrity of lysosomes. The NRU test is commonly used for the determination of cell viability based on the pH-sensitive accumulation of Neutral Red dye in lysosomes. Under physiological pH conditions, this cationic dye crosses the cell membrane by passive diffusion and binds to negatively charged components inside lysosomes, thereby reflecting lysosomal integrity and cellular viability [55]. In the present study, the results of this assay (Figure 7) showed a dose-dependent decrease in NRU levels, with the most pronounced effect observed at 500 µg/mL: in A375 cells, the NRU level was 65.61%, whereas in RPMI 7951 cells, it was 68.95%. Lysosomes play a key role in the growth, proliferation, and metastatic progression of melanoma. Through the autophagy mechanism, lysosomes help tumors adapt to their environment, which is commonly defined by nutrient deprivation and oxidative stress. Also, the Ca^2+^ lysosomal channel mucolipin 1 (MCOLN1) enhances melanoma cell survival and proliferation due to alterations in signaling pathways like MAPK and mTORC1. Moreover, the activity of transcription factors, for example, MITF (microphthalmia-associated transcription factor), is associated with lysosome- and endolysosome-related genes. MITF dysregulation and alteration of lysosome pathways lead to increased tumor growth and development in melanomas [56]. Also, melanoma lysosomes have a role in the development of resistance to different types of therapy like chemotherapy, targeted therapy, and immunotherapy [57,58,59]. Taking into account all these factors, assessment of the functioning of lysosomes is very important during the evaluation of cytotoxicity of extracts or other substances on melanoma models.

Considering the dose-dependent cytotoxicity, the changes in morphology and the integrity of lysosomes, further studies were carried out in order to estimate the possible effect of Ej extract on the oxidative status of cancer cells. Oxidative stress is known to be one of the major factors that contribute to cancer cell death. For this reason, intracellular reactive oxygen species (ROS) were estimated in melanoma cell lines after 48 h of treatment. As seen in Figure 8A, the level of intracellular ROS increased in RPMI 7951 cells from 98.35% at 50 µg/mL to 165.06% at 500 µg/mL. A similar trend was observed in A375 cells (Figure 8B), where ROS production increased from 95.02% to 149.09% across the same concentration range. These results indicate a clear dose-dependent (Figure 8) induction of oxidative stress in both melanoma cell lines. Reactive oxygen species are known to play a dual role in cancer biology, being involved in both tumor progression and cell death [60]. Nonetheless, excessive accumulation of ROS may impair cellular redox balance, resulting in oxidative damage and triggering apoptotic mechanisms [61]. Consequently, the increased amount of ROS found in the present study may be linked to the decrease in viability and morphological changes in cell lines previously mentioned. Moreover, increased levels of intracellular ROS have been strongly linked to mitochondrial dysfunction and apoptosis induction and may reflect oxidative stress as a possible mechanism of cytotoxicity in this case [62]. On the other hand, it is known from the literature that an increase in ROS levels after treatment of the cancer cell lines with the major components of the extract used in this study, such as chlorogenic acid, may be observed. In this way, in the study performed by Ranjbary et al., an elevated level of ROS was recorded after 24 h treatment of HT-29 colorectal cancer cells with 100 μM chlorogenic acid compared to the control group [63]. Likewise, Yang et al. showed that there was a significant increase in ROS levels after 24 h exposure to the same substance (50 to 200 μM) in the U937 leukemia cells [64]. Furthermore, a pro-oxidative activity of rutin was reported in HPV16-positive human cervical cancer CaSki cell line in terms of ROS production [65]. However, according to the available literature data, leaf extracts have been shown to provide protection against oxidative stress at low concentrations, which is in agreement with the results of this study at the concentration of 50 μg/mL in both cell lines. For instance, Kwon et al. reported that Ej leaf extract demonstrated ROS scavenging activity and promoted the activation of antioxidant pathways, including AMPK/Nrf2, after 24 h of exposure to 10 μg/mL [66]. Overall, this concentration-dependent response may be associated with the hormetic behavior of the compounds present in the extract, a phenomenon widely documented for natural compounds in evaluations involving cell lines, occurring as an adaptive response to conditions of moderate stress [67].

Then, the study was additionally extended to the analysis of mitochondrial function, and the assessment of mitochondrial membrane potential (ΔΨm) by using JC-1 staining was the first step in the process. The mitochondrial membrane potential (ΔΨm) indicates the energy-generating capacity of mitochondria via oxidative phosphorylation, which decreases as one of the first signs of the beginning of the apoptotic process. This indicator is measured using the JC-1 dye that accumulates in mitochondria depending on their potential. Polarized mitochondria produce red-fluorescent complexes, while non-polarized mitochondria produce green fluorescence. Therefore, the red/green fluorescence ratio is a marker of the functionality of mitochondria [68]. In the present study, treatment of RPMI 7951 and A375 cells with Ej extract for 48 h resulted in a progressive, concentration-dependent decrease in mitochondrial membrane potential, evidenced by a decrease in the JC-1 aggregate/monomer ratio being at 500 µg/mL 35.69% for RPMI 7951 (Figure 9) cells and 52.29% for A375 cells (Figure 10). Such ΔΨm alterations are indicative of malfunctioning mitochondria, which have been shown to play an important role in the induction of programmed cell death. Hence, the observed ΔΨm dysregulation is consistent with the early events of the mitochondrial apoptotic-like pathway; however, apoptosis was not directly confirmed in the present study, and this interpretation remains suggestive [69]. However, assessing mitochondrial function in melanoma studies can represent an important step due to its involvement in the pathogenesis and molecular mechanisms underlying melanoma, while also maintaining an environment favorable for malignant cell growth. Thus, in melanoma cells, an increase in mitochondrial oxidative phosphorylation is typically observed, leading to enhanced ATP production and thereby supporting cellular proliferative processes. On the other hand, mitochondria are a major source of reactive oxygen species, and at moderate levels, these molecules play a role in activating signaling pathways involved in oncogenesis, such as the MAPK pathway, as well as in promoting pro-survival autophagy under hypoxic or unfavorable conditions [70]. In addition, the role of mitochondria in resistance to antimelanoma therapies, particularly to BRAF inhibitors, is well documented [71]. Similar results to our findings have been reported for an Ej leaf extract in human promyelocytic leukemia cells (HL-60) [72]. Considering the changes in ΔΨm, the effect of the Ej extract on the mitochondrial network morphology was subsequently investigated using the MitoTracker CMXRos assay, which evidenced that morphological changes, along with chromatin condensation, were most pronounced at concentrations of 400–500 μg/mL in both cell lines, although a more pronounced effect was observed in the RPMI 7951 cell line (Figure 11).

Increased intracellular ROS levels together with the observed loss of mitochondrial membrane potential suggest the involvement of mitochondrial dysfunction in the cytotoxic effects induced by the Ej extract. In cancer cells, treatment-induced nuclear alterations, such as chromatin condensation and nuclear fragmentation, are widely recognized as apoptosis-associated morphological features and reflect changes in chromatin organization and genomic integrity [73]. Accordingly, the nuclear changes observed here are suggestive of an apoptotic-like process. The results obtained following Hoechst 33342 and MitoTracker staining revealed a concentration-dependent progression of cellular stress in both RPMI 7951 (Figure 11) and A375 (Figure 12) melanoma cells. At lower concentrations (50–100 µg/mL), cells maintained a morphology comparable to untreated controls, with preserved nuclear and mitochondrial structures. However, beginning with medium concentrations (200–300 µg/mL), a reduction in cell confluency was noticed alongside chromatin condensation, changes in nuclear shape, and uneven mitochondrial fluorescence. These phenomena were even more pronounced when higher concentrations (≥400 µg/mL) were employed, where nuclear fragmentation, a reduced number of adherent cells, and disruption of the mitochondrial network were noticed. At 500 µg/mL, the observed changes were exacerbated, as evidenced by significant nuclear condensation and structural changes to the mitochondria indicative of mitochondrial damage.

Our results agree with the report of Uto et al., according to which four triterpenes, namely corosolic acid (CA), ursolic acid (UA), maslinic acid (MA), and oleanolic acid (OA), from the methanolic extract of Ej had pro-apoptotic properties in human leukemia cell lines [74]. Similar results were obtained after Ej leaf extract treatment in human promyelocytic leukemia cells (HL-60) [72].

The final part of the study focused on the evaluation of the safety profile of the Ej leaf extract by assessing the anti-irritant potential using the CAM assay. The Hen’s Egg Test-Chorioallantoic Membrane (HET-CAM) test is one of the most widely used tests for the assessment of the irritant nature of plant extracts due to numerous reasons. First, the test makes use of chorioallantoic membrane from fertilized hen eggs that are rich in blood vessels and react similarly to the human tissue, thus making it an adequate in vivo test [75]. Second, the test is both cheap and simple; moreover, the test helps to avoid the ethical issues of animal testing. Additionally, the quick result of the test adds to the practicality of the test during the development stage [76].

Our study involved the HET-CAM assay performed as a complementary test in ovo for the assessment of the local irritant nature of the Ej aqueous leaf extract. This model is particularly useful for screening vascular irritancy, as the CAM is highly vascularized and allows rapid visualization of hemorrhage, vascular lysis, and coagulation. In the present study (Figure 13), the Ej extract at 500 µg/mL yielded an IS value of 0.07, comparable to distilled water, whereas 1% SDS elicited a strong irritant response. These results indicate that the extract does not induce acute vascular irritation under the tested conditions. Importantly, the absence of CAM irritation should be interpreted together with the in vitro profile. At biologically active concentrations, the Ej extract reduced melanoma cell viability, impaired lysosomal function, increased ROS production, and altered mitochondrial membrane potential, particularly in RPMI 7951 and A375 cells. However, the HET-CAM result suggests that these antitumor-related cellular effects are not accompanied by nonspecific acute vascular irritation in the CAM model. This observation is also consistent with the comparatively moderate impact observed in HaCaT keratinocytes, supporting a preliminary favorable local tolerability profile. Nevertheless, HET-CAM evaluates acute irritancy only and does not replace extended toxicological studies; therefore, further investigations using higher concentrations, repeated exposure models, and additional normal skin-related systems are required to define the safety threshold of the extract.

### Study Limitations and Future Directions

Although the in vitro findings obtained in the melanoma cell lines demonstrated a cytotoxic effect, the present study should be regarded only as a preliminary descriptive evaluation of the cytotoxic potential of Ej extract against melanoma cells after 48 h of treatment within the selected concentration range. Accordingly, several limitations should be acknowledged and addressed in future investigations. First, the study was performed exclusively using two human 2D melanoma cell lines (RPMI-7951 and A375). Accordingly, future research should expand on this work by assessing the effects of the extract in more advanced 3D models such as multicellular spheroids, organoids, or tissue-engineered models. Moreover, the concentration and exposure time should be varied in order to fully understand the biological activity of the extract. It is important to note that the 48 h time frame captures an early cytotoxic response, given that melanoma cells may partially recover by proliferation following short-term treatment. Therefore, further studies with longer exposure times and washout/recovery phases are needed to establish the durability of the observed effect. Second, this current research has not identified any of the molecular mechanisms responsible for the cytotoxicity effects seen. In particular, although the observed morphological and mitochondrial changes are suggestive of an apoptotic-like process, apoptosis was not directly confirmed; future work should include specific apoptosis assays (e.g., Annexin V/PI staining, caspase-3/7 activation, or PARP cleavage) to establish the precise mode of cell death. Consequently, future studies should focus on elucidating the signaling pathways and molecular targets involved in the extract’s activity, including the MAPK/ERK and PI3K/Akt/mTOR pathways, as well as the impact on regulators of epithelial–mesenchymal transition (EMT), metastasis, and apoptosis. In addition, tests to elucidate the biological mechanisms of action were performed only at the level of tumor cell lines, since the HaCaT cell line did not show a significant decrease in cell viability and no significant morphological changes were observed. Thus, subtle subcytotoxic effects of the extract at the level of healthy keratinocyte cells cannot be excluded, and the selectivity reported in this study refers to the final criteria of cell viability and morphology. Future studies are needed to expand the mechanistic panel to non-tumor cells.

Another limitation concerns the botanical source of the plant material used in this study. Leaves were collected from a single tree at a single time point in October 2024, and the phytochemical data presented characterize this specific plant material and batch of extractives. Because parameters such as phenolics and antioxidants of *E. japonica* leaves may vary with leaf age, harvest season, and cultivation conditions, the data reported in this study should be interpreted in the context of the harvest described. Therefore, future studies involving multiple harvests at different times of the year from multiple trees are needed to provide a more comprehensive description of the seasonal phytochemical profile. A final limitation concerns the choice of non-tumor control cells, HaCaT. HaCaT keratinocytes, although representing the normal cellular environment of the epidermis, are not compatible with melanoma cell lines, and normal human epidermal melanocytes were not included in this study. Therefore, the preferential effect reported in the present study should be interpreted as relative selectivity over non-tumor keratinocytes, not as cell line-specific selectivity over transformed melanocytes. It should also be noted that HaCaT cells, although non-tumor, are spontaneously immortalized and carry UV-type TP53 mutations and therefore do not show the full profile of normal primary keratinocytes [77]. Because melanocytes in vivo depend on interaction with surrounding keratinocytes, future studies should utilize either keratinocyte–melanocyte co-cultures or reconstructed human epidermis models, rather than melanocyte monocultures, to assess cell line-specific selectivity under conditions as close to physiological as possible [78].

## 4. Materials and Methods

### 4.1. Chemicals

The solvents used to perform LC/MS analysis were ultrapure water, methanol, and formic acid from WVR Chemicals (International Europe Services S.R.L., Bucharest, Romania). Labeled standards were purchased from Toronto Research Chemicals (Toronto, ON, Canada), as follows: Chlorogenic acid 13C3 (TRC-C366542), trans-p-Coumaric-d6 Acid (C755392), Rutin-d3 (R701800), 3,4-Dihydroxybenzoic Acid-d3 (TRC-D451682), Caffeic Acid-13C3 (C080002), Ferulic Acid-d3 (F308902), Quercetin-d3 (Q509502), Genistein d4 (G350010), and Resveratrol 13C6 (R150002) and Gallic acid D2 (DRE-C13998281) from Dr. Ehrenstorfer. Native compounds were bought from the same manufacturers in order to perform the calibration curve: trans-p-coumaric acid (DRE-C11734100), Chlorogenic acid (DRE-C11415750), Rosmarinic acid (DRE-C16819500), Caffeic acid (DRE-C10934700), trans-Ferulic acid (DRE-C13644100), Gallic acid (DRE-C13998280), (E/Z)-Ferulic acid (DRE-C13644090), Rutin (DRE-C16880000), 3,4-Dihydroxybenzoic acid (DRE-C12634710), Quercetin (DRE-C16695000), and Genistein (DRE-C13999800) from Dr. Ehrenstorfer, and 3,5-Dihydroxybenzoic Acid (D451700), 2,4-Dihydroxybenzoic Acid (D678940), Resveratrol (R150000), and p-coumaric acid (C755365) from Toronto Research Chemicals.

The chemicals used for the total phenolic content (TPC) determination included Folin–Ciocalteu reagent (Merck KGaA, Darmstadt, Germany) and sodium carbonate (Na_2_CO_3_) (Sigma-Aldrich Chemie GmbH, Buchs, Switzerland). The reagents used for the CUPRAC assay included neocuproine (GR for analysis, Merck KGaA, Darmstadt, Germany), copper(II) chloride dihydrate (CuCl_2_·2H_2_O, Merck KGaA, Darmstadt, Germany/Sigma-Aldrich Chemie GmbH, Buchs, Switzerland), ammonium acetate (Sigma-Aldrich Chemie GmbH, Buchs, Switzerland), and Trolox [(+/−)-6-hydroxy-2,5,7,8-tetramethylchroman-2-carboxylic acid, purum, >98.0% HPLC, Sigma-Aldrich Chemie GmbH, Buchs, Switzerland].

For the in vitro investigations, the following reagents were employed: phosphate-buffered saline (PBS), dimethyl sulfoxide (DMSO), Eagle’s Minimum Essential Medium (EMEM), Dulbecco’s Modified Eagle Medium (DMEM), penicillin/streptomycin solution, and fetal bovine serum (FBS), all procured from ATCC (American Type Culture Collection, Manassas, VA, USA). The MTT reagent, namely 3-(4,5-dimethylthiazol-2-yl)-2,5-diphenyltetrazolium bromide, was supplied by Roche Holding AG (Basel, Switzerland). Hoechst 33342 fluorescent nuclear dye and MitoTracker Red CMXRos were acquired from ThermoFisher Scientific (Waltham, MA, USA), while the JC-1 mitochondrial membrane potential assay kit was purchased from Elabscience (Houston, TX, USA). Neutral red reagent was obtained from Merck KGaA (Darmstadt, Germany). A 4% paraformaldehyde solution prepared in PBS was sourced from Santa Cruz Biotechnology (Dallas, TX, USA). Intracellular oxidative stress was assessed using ROS-Glo™ H_2_O_2_ assay kits purchased from Promega Corporation (Madison, WI, USA).

### 4.2. Plant Material and Aqueous Extract Preparation

**Figure 14:**
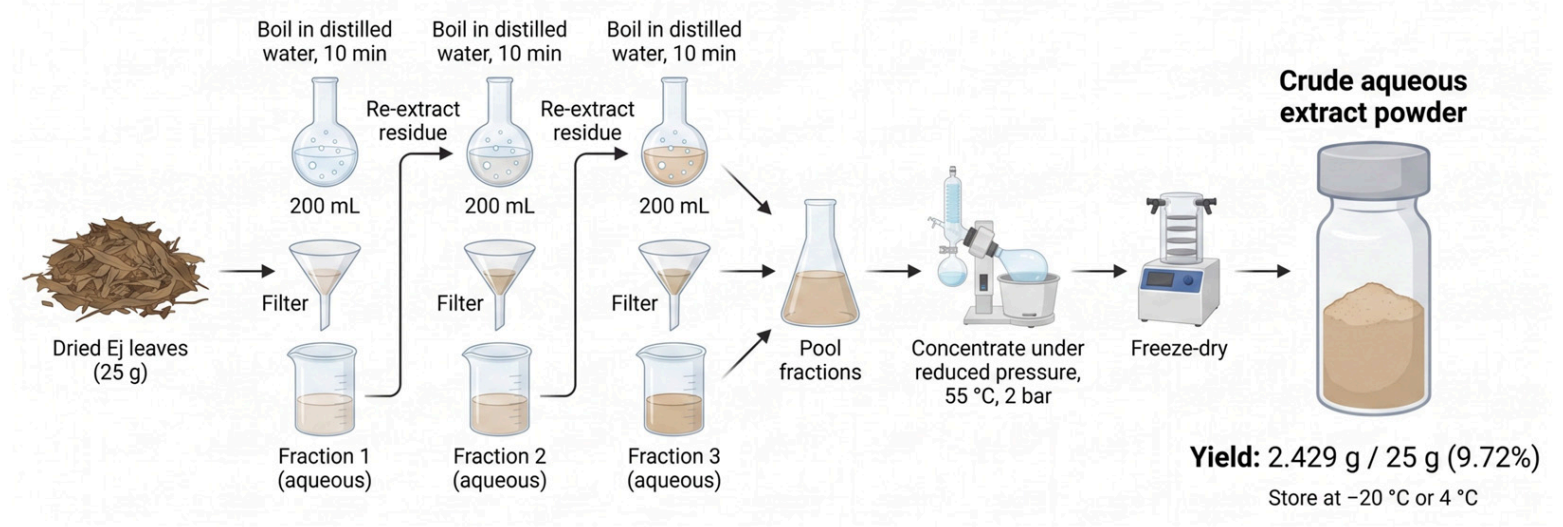
Schematic illustration of the process for preparing the aqueous extract of Ej leaves. Created in BioRender. Cristea, A. (2026) https://BioRender.com/pg52o0o.

The aqueous extract was prepared using leaves of Ej (Figure 14), which were collected from the Botanical Garden of “Victor Babeș” University of Medicine and Pharmacy from Timisoara (UMFVBT), from a single tree. The plant material, approximately 2 kg of leaves, was collected on the same day, in October 2024. The identity of the plant material was confirmed at the Department of Pharmaceutical Botany by Prof. Tchiakpe-Antal D.S., and a voucher specimen was deposited at the UMFVBT Herbarium (reference nr. CA_Ej_01). The leaves were dried at room temperature, protected from light, and finely ground for extraction, resulting in approximately 250 g of dry powder. Of this total, 25 g were used for extract preparation. All experiments were performed with a single batch of extract.

The Ej aqueous extract was composed of 3 extraction fractions obtained as follows: (1) fraction 1 was prepared by soaking 25 g of dried plant material (ground Ej leaves) in 200 mL of boiling distilled water for 10 min and filtering using a Whatman paper nr. 4; (2) fraction 2 was prepared by subjecting the resulting vegetal material to another extraction in 200 mL boiling water for 10 min; and (3) fraction 3 was prepared using the plant residue obtained after the second filtration, which was subsequently subjected to a third extraction with 200 mL boiling water. Each time, the duration of the extraction was 10 min. The three aqueous extracts separated by filtration were combined and concentrated under reduced pressure at 55 °C and 2 bar. Afterward, the concentrated extract was freeze-dried in order to obtain a dried, powdery extract. This powdery extract, representing the crude water extract of Ej leaves, was stored at −20 °C/4 °C until further use in tests. The freeze-drying process yielded 2.429 g of dried extract from 25 g of dried leaves, corresponding to an extraction yield of 9.72%.

### 4.3. Physicochemical Characterization of the Extract

#### 4.3.1. Total Polyphenol Content (TPC) Determination

To determine TPC, the Folin–Ciocalteu method was applied, as described in the protocol presented by Bordean et al. (2019) [79]. A volume of 0.5 mL of extract was mixed with 2.5 mL of Folin–Ciocalteu reagent (diluted 1:10), then 2 mL of 7.5% Na_2_CO_3_ (sodium carbonate) solution was added. The mixture was incubated for 2 h at room temperature, and the absorbance was read spectrophotometrically at 750 nm. A calibration curve was created for gallic acid as standard, and the results were expressed as mg gallic acid equivalent per gram of dry extract (mgGAE/100 g). All measurements were performed in triplicate.

#### 4.3.2. Determination of Polyphenol Profile by LC-MS Analysis

To identify and quantify polyphenols, a Shimadzu (Shimadzu Corporation, Kyoto, Japan) ultra high-performance liquid chromatographic (UHPLC) equipment coupled with an LCMS 8045 triple quadrupole mass spectrometer (Shimadzu Corporation, Kyoto, Japan) was used. The specific configurations of the system include LC-30AD × 2 solvent delivery pumps, DGU-20A5R degassing unit, CTO-20AC column oven, SIL-30AC autosampler, CBM-20A system controller, and the identification was performed with an 8045 triple quadrupole mass spectrometer. The results were analyzed using the LabSolutions Ver. 5.86 software. For this purpose, a volume of 5 µL of Ej extract was injected, and the polyphenols were separated using a C18 reversed-phase column (2.7 µm, 2.1 × 100 mm) from Shimadzu (Kyoto, Japan). Prior to injection, the reconstituted extract was diluted [1:10] with water: methanol to bring analyte concentrations within the calibration range; reported concentrations were back-calculated using the corresponding dilution factor. All quantified analytes fell within the calibrated range (50–5000 ng/mL) in the injected solution. Mobile phase A consists of ultrapure water with 0.05% formic acid solution (*V*/*V*). Mobile phase B consists of methanol with 0.05% formic acid solution (*V*/*V*). The gradient followed the following proportions: the start is with 10% methanol, up to 5 min followed by 95% methanol up to 8 min, and 10% methanol, up to 14 min, with a flow rate of 0.4 mL/min and a temperature of 40 °C. MS detection used an electrospray ionization (ESI) source. Thus, the lyophilized extract was dissolved in a mixture of water and methanol, supplemented with an internal standard, and analyzed. External standards were used to quantify identified compounds, with calibration curves comprising 50 ng/mL to 5000 ng/mL, presenting good linearity (R2 > 0.998). The limit of quantification was 50 ng/mL, and the limit of detection (LOD) was 15 ng/mL. With regard to the precision of the method, an average RSD (%) value of less than 15% is generally considered an acceptable range to validate quantitative analytical methods, indicating good accuracy and precision [20].

#### 4.3.3. Total Antioxidant Capacity Analysis

Total antioxidant capacity was assessed according to the protocol described by Bordean (2016), following the CUPRAC method [80]. Briefly, the Ej leaf extract was exposed to a solution of copper chloride (CuCl_2_), neocuproin, and an ammonium acetate buffer. The mixture was incubated for 30 min, and the absorbance was measured at a wavelength of 450 nm using a T60 UV-Visible Spectrophotometer (PG Instruments Ltd., Lutterworth, UK). The quantitative determination was performed using Trolox as a reference standard, and the results were expressed as µmol Trolox equivalents per gram of dry matter (µmol TE/g). The determinations were performed in triplicate [81].

### 4.4. Cell Lines and Culture Conditions

This study was conducted using two human melanoma cell lines obtained from the American Type Culture Collection (ATCC, Manassas, VA, USA): RPMI 7951 (HTB-66™) and A375 (CRL-1619™), as well as the normal human keratinocyte cell line HaCaT (300493), purchased from Cell Lines Service (CLS, Eppelheim, Germany). The cell lines were cultured in specific growth conditions, as follows: RPMI 7951 required Eagle’s Minimum Essential Medium (EMEM) supplemented with 10% fetal bovine serum (FBS) and 1% antibiotic mixture (100 U/mL penicillin and 100 μg/mL streptomycin), whereas A375 and HaCaT cells were cultured in Dulbecco’s Modified Eagle Medium (DMEM) supplemented with 10% FBS and 1% penicillin–streptomycin. All cell cultures were incubated at 37 °C in a humidified atmosphere containing 5% CO. For the cytotoxicity assay, all cell culture procedures were performed under aseptic conditions. Thus, the cells were treated with the Ej extract at concentrations ranging from 50 to 500 µg/mL, prepared from a 100 mg/mL stock solution in sterile DMSO. The working solutions were obtained by further dilution of the stock solution in sterile culture medium specific for each cell line. Throughout the experimental period, the cell cultures were regularly examined macroscopically and microscopically for signs of microbial contamination. These approaches are commonly used for routine monitoring of cell cultures, as bacterial contamination may be indicated by visible changes in the culture medium and may also be detected by microscopic examination [82,83], and no signs of contamination were observed during the experiments.

For a more comprehensive study at the melanoma level, two complementary tumor cell lines were selected. On the one hand, the A375 cell line (ATCC CRL-1619) derived from a primary amelanotic cutaneous melanoma and with wild-type TP53. On the other hand, the RPMI 7951 cell line (ATCC HTB-66) derived from a lymph node metastasis and with the presence of a homozygous nonsense TP53 mutation and homozygous PTEN loss [84]. Although both cell lines carry the BRAF V600E mutation, PTEN loss is an important factor regarding the efficacy of BRAF-targeted therapy [85]. For this reason, the evaluation of the extract at the level of both melanoma cell types provides information regarding its efficacy at distinct stages of the disease and in different genetic contexts relevant for the response to therapy. Human HaCaT keratinocytes were used as control, non-tumor cells, because they constitute the normal cellular environment surrounding melanocytes within the epidermal melanin unit [78] and are the predominant cell type that is exposed to a topically applied pharmaceutical preparation. In addition, HaCaT are spontaneously immortalized, without viral oncogenes [86], growing under the same culture conditions as the melanoma cells used in the present study and exhibiting a proliferation rate comparable to them [87]. For these reasons, variability factors related to culture conditions and proliferation rate are minimized. On the other hand, normal human epidermal melanocytes require special culture media, enriched with growth factors and, frequently, phorbol esters that directly modulate cell signaling [88]. In addition, they may exhibit slow proliferation and marked donor-dependent variability.

### 4.5. Cellular Morphology Assessment Using Bright-Field Microscopy

The morphological changes induced by Ej extract at the tested concentrations (50–500 µg/mL) after 48 h of treatment were examined in RPMI 7951, A375, and HaCaT cells. Representative images of control (untreated cells) and Ej-treated cells were captured under bright-field conditions using a Lionheart FX automated microscope (BioTek Instruments Inc., Winooski, VT, USA). Image acquisition and processing were performed with Gen5™ Microplate Data Collection and Analysis Software (Version 3.14, BioTek Instruments Inc., Winooski, VT, USA).

### 4.6. Cell Viability Assessment Using the MTT Assay

The MTT assay was conducted to evaluate the impact of Ej aqueous extract on cell viability. The experimental protocol was established based on the literature data and adapted to our lab conditions [89]. Briefly, RPMI 7951, A375, and HaCaT cells were seeded at a cell density of 1 × 10^4^ cells/well in 96-well plates and allowed to reach the desired confluence (approximately 70%). The cells were then treated with increasing concentrations of Ej extract—50, 100, 200, 300, 400, and 500 µg/mL—for 48 h (the dilutions were prepared from a 100 mg/mL stock solution of the lyophilized aqueous Ej extract in DMSO. In all tested concentrations, the final DMSO concentration did not exceed 0.5%). After the treatment period, the culture medium was replaced with fresh medium, and 10 µL of MTT solution was added to each well. After 3 h of incubation at 37 °C, 100 µL of MTT solubilization solution was added to each well, and the plates were kept at room temperature and in the dark for 30 min to ensure complete dissolution of the formazan crystals. Finally, the absorbance was measured at 570 nm, with background correction at 630 nm, using a microplate reader Cytation 5 from BioTek Instruments, Winooski, VT, USA [90]. In compliance with the ISO 10993-5:2009 guidelines [91] and OECD recommendations for cytotoxicity assays, the concentrations of test compounds that maintain ≥70% cell viability are considered not to exhibit cytotoxic effects. The 48 h exposure period was selected based on the interval used in the NCI-60 in vitro screening panel for potential anticancer agents [92] and protocols previously validated by our working group [9,67,89]. This interval was also based on the proliferation characteristics of the two cell lines, representing approximately two population doublings for A375 and one population doubling for RPMI 7951 cells, allowing the evaluation of all criteria at the same time and favoring direct comparison between cytotoxic and mechanistic parameters [93,94]. In addition, because polyphenols can undergo modifications by autoxidation with the generation of hydrogen peroxide and other residual substances that can accumulate with prolonged incubation, the exposure period was limited to 48 h [95].

### 4.7. Cytotoxicity Evaluation by Neutral Red Uptake Assay (NRU)

The cytotoxicity of the Ej extract was further assessed using the Neutral Red Uptake assay. Thus, RPMI 7951 and A375 cells were seeded in 96-well flat-bottom plates at a density of 1 × 10^4^ cells per well and allowed to adhere under standard culture conditions. After attachment, the cells were treated for 48 h with increasing concentrations of Ej extract (50, 100, 200, 300, 400, and 500 µg/mL). At the end of the exposure period, the culture medium was removed and replaced with 100 µL per well of Neutral Red working solution (40 µg/mL NR prepared in complete culture medium). To facilitate dye uptake, the plates were incubated for 2 h at 37 °C in a humidified atmosphere containing 5% CO_2_. Then, excess dye was removed by washing two times with PBS. After that, representative images were acquired under bright-field illumination using a Lionheart FX automated microscope (BioTek Instruments Inc., Winooski, VT, USA). For dye extraction, 150 µL per well of NR destaining solution consisting of 50% ethanol, 49% deionized water, and 1% glacial acetic acid was applied. Following this, the plates underwent gentle shaking for 10 min to allow complete release of the intracellular dye. Finally, absorbance values were registered at 540 nm utilizing the Cytation 5 multimode microplate reader [96].

### 4.8. Intracellular ROS Determination

Intracellular ROS generation in RPMI 7951 and A375 cells was assessed using the ROS-Glo H_2_O_2_ Assay (Promega, Madison, WI, USA). The experimental procedure followed the manufacturer’s instructions. The cells were seeded at a density of 1 × 10^4^ cells per well in 96-well opaque white plates and allowed to adhere. Subsequently, the cells were treated with different concentrations of Ej (50, 100, 200, 300, 400, 500 µg/mL) for 48 h. After 42 h of incubation, a volume of 20 μL of ROS-Glo™ H_2_O_2_ substrate solution, prepared according to the manufacturer’s protocol, was added to each well. The plates were then returned to standard culture conditions (37 °C, 5% CO_2_) for the remaining treatment period. At the end of the 48 h incubation period, 100 μL of ROS-Glo™ detection reagent was added to each well, followed by a 20 min incubation at room temperature in the dark. Luminescence was measured using the Cytation 5 multimode reader (BioTek Instruments, Winooski, VT, USA). Data processing was performed according to the method described by Feher et al. [97].

### 4.9. Assessment of Mitochondrial Membrane Potential (ΔΨm)

Changes in the mitochondrial membrane potential in RPMI 7951 and A375 melanoma cells after 48 h of exposure to Ej extract (50, 100, 200, 300, 400, and 500 µg/mL) were investigated using the JC-1 fluorescent probe in accordance with the protocol described by Talpoș et al. [67]. The cells were seeded in black-walled, clear-bottom 96-well plates at a density of 1 × 10^4^ cells per well and allowed to attach until approximately 70% confluence was reached. The cells were then treated with Ej extract and incubated for 48 h at 37 °C in a humidified atmosphere containing 5% CO_2_. At the end of the treatment period, the culture medium was carefully removed, and the cells were gently washed with PBS. Subsequently, 100 µL/well of JC-1 staining solution (5 µM) was added, and the plates were incubated for 45 min at 37 °C under standard culture conditions. After staining, the cells were rinsed twice with PBS to eliminate excess dye. Fluorescence images were captured using the Lionheart FX automated microscope and further processed with Gen5™ Microplate Data Collection and Analysis Software. Red and green fluorescence intensities were measured at 590 nm and 529 nm, respectively, using the Cytation 5 multimode microplate reader (BioTek Instruments, Winooski, VT, USA).

### 4.10. Nuclear and Mitochondrial Integrity Evaluation

Mitochondrial and nuclear appearance after Ej extract treatment was examined in RPMI 7951 and A375 melanoma cells. The cells were seeded in 12-well plates at a 1 × 10^5^ cells/well cell density and allowed to attach until reaching the appropriate confluence. Subsequently, the cells were exposed for 48 h to Ej extract (50, 100, 200, 300, 400, and 500 µg/mL). First, the mitochondrial architecture was evaluated using MitoTracker Red CMX Ros. The MitoTracker dye was prepared as a 1 mM stock solution in DMSO and further diluted in the corresponding complete culture medium (EMEM supplemented with 10% FCS and 1% penicillin–streptomycin for RPMI 7951 and DMEM supplemented with 10% FCS and 1% penicillin–streptomycin for A375 cells) to a final concentration of 300 nM. The cells were incubated with the staining solution for 30 min at 37 °C under standard culture conditions, followed by washing with fresh culture medium to remove unbound dye. After MitoTracker labeling, the cells were fixed with 4% paraformaldehyde for 10 min at room temperature to preserve cellular morphology and then thoroughly rinsed with PBS. Next, nuclear morphology was assessed using Hoechst 33342. Thus, after fixation, the cells were exposed to a 1:2000 solution of Hoechst 33342 in PBS, which was left in contact with the cells in the dark for 5–10 min. The staining solution was subsequently removed, and the wells were washed three times with PBS. Representative fluorescence images were acquired using the Lionheart FX automated microscope at 20× magnification. Image processing and analysis were carried out using Gen5™ Microplate Data Collection and Analysis Software (version 3.14; BioTek Instruments Inc., Winooski, VT, USA) [98,99].

### 4.11. Quantitative Image Analysis

For quantification of the images obtained after immunofluorescence staining, Image J/Fiji software (v1.54, National Institutes of Health, Bethesda, MD, USA) was used. For this purpose, each image was spatially calibrated using its own scale bar, and individual channels were separated before analysis. For quantification of nuclei, a fixed intensity threshold was established, internal holes were filled, and adjacent nuclei were separated using the watershed function. For counting nuclei, the Analyze Particles function was applied, accepting objects with a size between 20 and 800 μm^2^ and excluding particles in contact with the edges of the image. Nuclear density was expressed as the number of nuclei per mm^2^ of analyzed field. For the mitochondrial signal, the same commands were run, and the labeled area was measured as a percentage of the field occupied by the MitoTracker-positive signal. Quantification was limited to count- and area-based metrics, without dependence on absolute signal intensity.

### 4.12. In Ovo Irritation Assay

In ovo irritation potential (as part of the safety profile) of the Ej leaf extract was assessed using the hen’s egg test on the chorioallantoic membrane (HET-CAM), following the ICCVAM-recommended protocol with minor adaptations [100,101]. Fertilized hen eggs were incubated for 9 days at 37 °C and 70% relative humidity. On embryonic day 4, approximately 9 mL of albumen was removed using a sterile syringe, and on day 5, a small window was created in the eggshell to allow direct visualization of the developing CAM; the window was subsequently sealed with medical tape. On embryonic day 9, 600 µL of Ej leaf extract at 500 µg/mL was applied directly onto the CAM. Distilled water was used as the negative control, while 1% sodium dodecyl sulfate (SDS) served as the positive irritant control. The CAM was monitored for 300 s using a Zeiss SteREO Discovery.V8 stereomicroscope coupled to a Zeiss Axiocam 105 digital camera (Carl Zeiss, Oberkochen, Germany). The onset times of hemorrhage (tH), vascular lysis (tL), and coagulation (tC) were recorded in seconds. The irritation score (IS) was calculated according to the following equation:

$$IS=5\times \left[\frac{301-Sec\;H}{300}\right]+7\times \left[\frac{301-Sec\;L}{300}\right]+9\times \left[\frac{301-Sec\;C}{300}\right]$$

When no vascular reaction IS = 5 × [(301 − Sec H)/300] + 7 × [(301 − Sec L)/300] + 9 × [(301 − Sec C)/300] was observed within the 300 s observation period, the endpoint time was recorded as 300 s. The samples were classified according to Luepke’s scale as non-irritant (IS = 0–0.9), mildly irritant (IS = 1–4.9), moderately irritant (IS = 5–8.9), or strongly irritant (IS = 9–21) [23].

### 4.13. Statistical Analysis

The statistical analysis of experimental data was performed using GraphPad Prism (version 10.2.3). All quantitative results obtained from the in vitro experiments were analyzed by one-way analysis of variance (ANOVA), followed by Dunnett’s multiple comparison test to compare treated groups with the control (untreated cells). Experiments performed on HaCaT cells served as a non-tumor reference. All assays were performed in three independent experiments, each in technical triplicate. Statistical significance was expressed as follows: ns, not significant (*p* ≥ 0.05); * *p* < 0.05; ** *p* < 0.01; *** *p* < 0.001; **** *p* < 0.0001 [102].

## 5. Conclusions

In conclusion, we demonstrated that the aqueous leaf extract of *Eriobotrya japonica* cultivated in Romania has a complex phytochemical composition characterized by a high phenolic content and notable antioxidant capacity. Moreover, the Ej extract reduced the viability of both RPMI 7951 and A375 melanoma cells in a concentration-dependent manner, while producing only limited effects in HaCaT keratinocytes. The cytotoxic mechanism of action included increased ROS generation, loss of mitochondrial membrane potential, alterations in lysosomal function, and marked mitochondrial and nuclear changes, indicating the involvement of mitochondrial dysfunction in the cytotoxic response. In addition, the extract did not induce irritation in the HET-CAM model, suggesting good local tolerability. These findings augment our comprehension of the antimelanoma potential of Ej leaf extract.

## Figures and Tables

**Table 1:** Concentration of the polyphenolic compounds identified in the Ej aqueous leaf extract by UHPLC-MS/MS analysis.

Compounds	Retention Time (RT)	Molecular Formula	Concentration [µg/mL]	*m*/*z* Experimental	*m*/*z* Calculated	MS/MS Fragments	Proposed Compound
1	2.62	C_16_H_18_O_9_	47.31 ± 0.01	353.087	353.1000	191.2000	Chlorogenic acid
2	5.28	C_27_H_30_O_16_	1.25 ± 0.40	609.140	609.1500	300.1000	Rutin
3	1.18	C_7_H_6_O_4_	0.89 ± 0.38	153.02	153.0300	108.9000	3,5-Dihydroxybenzoic Acid/ 3,4-Dihydroxybenzoic Acid
4	4.53	C_9_H_8_O_3_	0.78 ± 0.28	163.04	163.0500	119.0500	p-Coumaric acid/trans-p-Coumaric acid
5	2.75	C_7_H_6_O_4_	0.23 ± 0.01	153.02	153.0300	108.9000	2,4-Dihydroxybenzoic Acid
6	3.03	C_9_H_8_O_4_	0.11 ± 0.04	179.0	179.0400	135.0500	Caffeic acid
7	4.91	C_10_H_10_O_4_	0.10 ± 0.03	193.05	193.0600	134.1000	(E/Z)-Ferulic acid/trans-Ferulic acid
8	5.90	C_15_H_10_O_7_	<LOD	301	301.0400	151.1000	Quercetin
9	-	C_18_H_16_O_8_	NF	359	359.0800	161.1000	Rosmarinic acid
10	-	C_7_H_6_O_5_	NF	169.01	169.1000	125.0500	Gallic acid
11	-	C_14_H_12_O_3_	NF	227.07	227.0000	143.0000	Resveratrol
12	-	C_15_H_10_O_5_	NF	269.04	269.0000	133.0000	Genistein

Note: NF—not found (signal below LOD = 15 ng/mL); <LOQ—detected but below the limit of quantification (LOQ = 50 ng/mL); values are mean ± SD (n = 3). Isomeric pairs (3,5-/3,4-dihydroxybenzoic acid; p-coumaric/trans-p-coumaric acid; (E/Z)-/trans-ferulic acid) that could not be chromatographically resolved are reported in a single row.

**Table 2:** The signal-to-noise ratio (S/N) for compounds from the Ej extract.

Identified Compound Name	S/N
Chlorogenic acid	10.62
Rutin	41.42
3,5-Dihydroxybenzoic Acid/3,4-Dihydroxybenzoic Acid	41.26
p-Coumaric acid/trans-p-Coumaric acid	7.57
Caffeic acid	4.12
(E/Z)-Ferulic acid/trans-Ferulic acid	1.39
Quercetin	11.69

**Table 3:** Irritation scores of the Ej leaf extract and controls in the HET-CAM assay (n = 3).

Test Group	Hemorrhage (s)	Lysis (s)	Coagulation (s)	IS (Mean ± SD)	Luepke Classification
Ej extract (500 µg/mL)	>300	>300	>300	0.07 ± 0.00	Non-irritant
Negative control (distilled water)	>300	>300	>300	0.07 ± 0.00	Non-irritant
Positive control (1% SDS)	12	85	150	14.3 ± 2.0	Strongly irritant

Note: IS—irritability score, calculated according to Luepke [23]. Classification: non-irritant (IS = 0–0.9), mildly irritant (IS = 1–4.9), moderately irritant (IS = 5–8.9), strongly irritant (IS = 9–21). Hemorrhage, lysis, and coagulation times represent the onset of each endpoint during the 300 s observation period; “>300” indicates no reaction was observed within the observation window. Values are the mean ± SD (n = 3).

## Data Availability

The original contributions presented in this study are included in the article. Further inquiries can be directed to the corresponding author.

## Abbreviations

The following abbreviations are used in this manuscript:

AMPK/Nrf2	AMP-activated protein kinase/Nuclear factor erythroid 2–related factor 2
ATCC	American Type Culture Collection
ATP	Adenosine Triphosphate
BRAF	B-Raf Proto-Oncogene, Serine/Threonine Kinase
CA	Corosolic Acid
CAM	Chorioallantoic Membrane
CUPRAC	Cupric Ion Reducing Antioxidant Capacity
CuCl_2_	Copper(II) Chloride
CuCl_2_HO	Copper(II) Chloride Dihydrate
DMEM	Dulbecco’s Modified Eagle Medium
DMSO	Dimethyl Sulfoxide
Ej	*Eriobotrya japonica*
EMEM	Eagle’s Minimum Essential Medium
ESI	Electrospray Ionization
FBS	Fetal Bovine Serum
GAE	Gallic Acid Equivalents
HET-CAM	Hen’s Egg Test on the Chorioallantoic Membrane
IC_50_	Half-maximal inhibitory concentration
IS	Irritability Score
LC-MS	Liquid Chromatography–Mass Spectrometry
LOD	Limit of Detection
LOQ	Limit of Quantification
MAPK	Mitogen-Activated Protein Kinase
MCOLN1	Mucolipin-1 (Mucolipin Transient Receptor Potential Channel 1)
MITF	Microphthalmia-Associated Transcription Factor
mTORC1 MTT	Mechanistic Target of Rapamycin Complex 3-(4,5-dimethylthiazol-2-yl)-2,5-diphenyltetrazolium bromide assay
NRU	Neutral Red Uptake
OA	Oleanolic Acid
PBS	Phosphate-Buffered Saline
ROS	Reactive Oxygen Species
RSD	Relative Standard Deviation
SD	Standard Deviation
SDS	Sodium Dodecyl Sulfate
tC	Onset Time of Coagulation
TE	Trolox Equivalents
tH	Onset Time of Hemorrhage
tL	Onset Time of Vascular Lysis
TAC	Total antioxidant capacity
TPC	Total Phenolic Content
UA	Ursolic Acid
UHPLC	Ultra-High-Performance Liquid Chromatography
UPLC-QqQ-MS	Ultra-Performance Liquid Chromatography Triple Quadrupole Mass Spectrometry
